# Thermodynamics-Based Models of Transcriptional Regulation by Enhancers: The Roles of Synergistic Activation, Cooperative Binding and Short-Range Repression

**DOI:** 10.1371/journal.pcbi.1000935

**Published:** 2010-09-16

**Authors:** Xin He, Md. Abul Hassan Samee, Charles Blatti, Saurabh Sinha

**Affiliations:** 1 Department of Biochemistry and Biophysics, University of California, San Francisco, San Francisco, California, United States of America; 2 Department of Computer Science, University of Illinois at Urbana-Champaign, Urbana, Illinois, United States of America; Duke University, United States of America

## Abstract

Quantitative models of *cis*-regulatory activity have the potential to improve our mechanistic understanding of transcriptional regulation. However, the few models available today have been based on simplistic assumptions about the sequences being modeled, or heuristic approximations of the underlying regulatory mechanisms. We have developed a thermodynamics-based model to predict gene expression driven by any DNA sequence, as a function of transcription factor concentrations and their DNA-binding specificities. It uses statistical thermodynamics theory to model not only protein-DNA interaction, but also the effect of DNA-bound activators and repressors on gene expression. In addition, the model incorporates mechanistic features such as synergistic effect of multiple activators, short range repression, and cooperativity in transcription factor-DNA binding, allowing us to systematically evaluate the significance of these features in the context of available expression data. Using this model on segmentation-related enhancers in *Drosophila*, we find that transcriptional synergy due to simultaneous action of multiple activators helps explain the data beyond what can be explained by cooperative DNA-binding alone. We find clear support for the phenomenon of short-range repression, where repressors do not directly interact with the basal transcriptional machinery. We also find that the binding sites contributing to an enhancer's function may not be conserved during evolution, and a noticeable fraction of these undergo lineage-specific changes. Our implementation of the model, called GEMSTAT, is the first publicly available program for simultaneously modeling the regulatory activities of a given set of sequences.

## Author Summary

The development of complex multicellular organisms requires genes to be expressed at specific stages and in specific tissues. Regulatory DNA sequences, often called *cis*-regulatory modules, drive the desired gene expression patterns by integrating information about the environment in the form of the activities of transcription factors. The rules by which regulatory sequences read this type of information, however, are unclear. In this work, we developed quantitative models based on physicochemical principles that directly map regulatory sequences to the expression profiles they generate. We evaluated these models on the segmentation network of the model organism *Drosophila melanogaster*. Our models incorporate mechanistic features that attempt to capture how activating and repressing transcription factors work in the segmentation system. By evaluating the importance of these features, we were able to gain insights on the quantitative regulatory rules. We found that two different mechanisms may contribute to cooperative gene activation and that repressors often have a short range of influence in DNA sequences. Combining the quantitative modeling with comparative sequence analysis, we also found that even functional sequences may be lost during evolution.

## Introduction

Transcriptional gene regulation is largely achieved by binding of transcription factors (TFs) to their cognate sites in regulatory sequences (called binding sites), followed by interaction of the bound factors with the basal transcriptional machinery. Precise spatial-temporal patterns of a gene's expression, such as those seen for developmental genes, are the result of simultaneous action by a combination of TFs and their respective binding sites located within modular DNA segments called “*cis*-regulatory modules” (CRMs, also called enhancers). Tools of genetics and molecular biology have been used through years of painstaking experimentation to reveal examples of CRMs and their regulatory interactions with TFs [1]. Despite the empirical knowledge of such examples, our understanding of the rules by which various TFs, some activators and others repressors, work together to drive the precise expression pattern of a gene remains rudimentary.

Biochemical experiments [2] and genetic assays of synthetic CRMs [3], [4] have been two successful paradigms for exploring the mechanisms of transcriptional regulation. At the same time, there is widespread recognition [4] that such experimental paradigms need to be complemented with quantitative analyses, since the underlying rules of combinatorial regulation are themselves quantitative in nature. A quantitative model that relates regulatory sequences to their functional outputs [5], [6] can be a powerful tool in teasing out mechanistic insights from gene expression data. Additionally, it may allow us to predict the function of an uncharacterized piece of DNA, and may be harnessed to discover novel CRMs in a genome, as well as to predict the expression pattern driven by a known CRM in conditions where aspects of the input information differ from those in wild type.

The precise “quantitative modeling” problem we consider is the following: *given the sequence of a CRM, the concentration profiles (in space or time) of a set of transcription factors (TFs) and their respective DNA-binding specificities, predict the expression profile driven by the CRM, also called the “readout” of the CRM*. This expression profile can be tested experimentally by a reporter gene placed near the CRM. The quantitative model is the mathematical function that maps the input data to the CRM's readout. Such a model is typically based on the following, widely-accepted characteristics of the process of transcriptional regulation: (a) transcription factor (protein) molecules bind DNA, to an extent that depends on their concentration, binding specificity and the sequence of the binding site, and (b) gene expression (readout) depends on the combination of transcription factors bound to the DNA. The bound TF molecules act in concert to recruit the basal transcriptional machinery (BTM) to the promoter, thus initiating transcription [5].

Statistical thermodynamics provides a natural framework for quantitative models of transcriptional regulation, by modeling DNA binding and protein interactions in equilibrium conditions. In the theory laid out by Shea & Ackers and formalized by Buchler et al. [5], [7],

- Statistical thermodynamics (in particular, the Boltzmann distribution law) was used to compute the relative probability of every molecular configuration involving binding sites, transcription factors and the basal transcriptional machinery (BTM), and
- Gene expression was modeled as being proportional to the “fractional occupancy” [8] of the BTM at the promoter, i.e., the total probability of all configurations where the BTM is bound to the promoter.

This framework allows one to incorporate the competition between TFs for overlapping binding sites, as well as cooperative interactions between TFs bound at nearby sites. Sequence-specific TF-DNA binding can be incorporated into the framework as proposed by Berg & von Hippel [9], through the use of “position weight matrices” (PWMs) that represent the TFs' binding specificities [10].

In this work, we have developed and implemented quantitative models to predict expression from sequence, based on the statistical thermodynamics framework outlined above. Previous publications [6], [11]–[14] have adopted various aspects of the framework and applied them successfully to different gene expression data sets from yeast and fruit fly. However, most of these models cannot be applied to arbitrary sequences, or gloss over important mechanistic details such as the distinction between activator and repressor action (see below). To the best of our knowledge, the computational method we present here is the first implementation of the Shea-Ackers model that can be applied to any given sequence, with binding sites of varying affinities for their respective TFs. Furthermore, it models mechanistic details of activation and repression that were missing in the original Shea-Ackers model (which was developed for prokaryotic gene regulation) and which we expect to be relevant in the context of metazoan regulatory systems. The method involves summing the relative probabilities of all possible molecular configurations of the DNA segment. Since strong as well as weak binding sites may be crucial for the readout of a CRM [12], [15], and since a CRM may harbor generous numbers of such sites [16], there are an enormous number of possible configurations, leading to a severe computational challenge. We meet this challenge by devising efficient (dynamic programming) formulations of all of our model calculations. We apply our models to existing expression data from *Drosophila* embryonic development, to investigate mechanistic aspects of transcriptional regulation in this system. By comparing how well different models or models with different parameter settings explain the data, we attempt to understand the importance of various aspects of the model in light of the available data.

The Shea-Ackers model was developed for prokaryotic gene promoters, and lacks certain mechanistic aspects that have been much debated in the context of metazoan regulatory systems. One such aspect is the mechanism of transcriptional inhibitors (commonly called “repressors”), where several different possibilities have been suggested. Gertz et al. [12] modeled the repressive action of a TF through direct destabilizing interactions with the BTM, while Janssens et al. [13] assumed a “quenching” mechanism where a bound repressor molecule shuts off activator binding within a limited distance, e.g., 100 bp, around itself [17], [18]. A third possible mode of repressor action is through direct competition with activating TFs for binding at overlapping sites, as suggested by the observation that activator and repressor sites often overlap [19]. In the segmentation system in *Drosophila*, existing experimental work on a few well-characterized or synthetic CRMs seemed to suggest that repressors act through the quenching, or short-range mechanism. However, it is not known whether this is true for all CRMs. Also, it is possible that the same repressor works though multiple mechanisms (e.g., *Kr*, a well known short range repressor [20] may also repress through interaction with BTM [21]). Here, we begin to address these questions by implementing all of the above modes of repressor action within a common framework, and allowing any of them to be used in fitting the model to available data. A significantly better agreement between data and model may then be interpreted as evidence in favor of the chosen model of repression, since all other aspects of the model remain fixed.

Another mechanistic question that has repeatedly surfaced in the study of metazoan regulation pertains to the role of multiple activator sites often present in the same regulatory sequence. One line of thought has been that this enables cooperative DNA-binding by multiple activator molecules [22], [23], i.e., DNA-binding of one activator molecule facilitates binding of other ones, and is key to achieving the highly non-linear response to an activator concentration gradient that underlies certain gene expression patterns in development [2]. However, such a non-linear response may also be achieved by another mechanism called “transcriptional synergy” [24]. If multiple activator molecules simultaneously interact with the BTM, the result may be a kind of synergistic activation where the activation effect of two binding sites is greater than the sum of each [25], even in the absence of DNA-binding cooperativity. Not only are these two mechanisms different biochemically, they respond differently to the change of TF concentrations [26]. (Also see Text S1 and Figure S1 for a discussion of how the two mechanisms affect transcriptional activation differently, using a sequence with multiple identical binding sites as an example.) Despite a number of experimental studies [2], [8], [27], [28], the relative importance of each is unclear and represents a major gap in our understanding of transcriptional regulation [26]. We implemented both modes of multi-activator synergy in our model. As with repressor action, we sought to assess their relative contributions systematically by testing which variant of the model agrees best with the data.

### Summary of results

We began by examining whether our models agree with existing data on transcriptional gene regulation during *Drosophila* embryonic development (anterior–posterior axis specification). This involved training our model on 37–44 experimentally characterized CRMs and 6–8 transcription factors. The overall quality of fit as well as predictive ability of our models was remarkably high. Next, we applied different model variants to investigate mechanistic questions. We found that the transcriptional synergy arising from simultaneous contact of activators with the BTM contributes significantly to the accurate specification of expression patterns, and this contribution extends beyond the contribution from mutual interactions (DNA-binding cooperativity) between activators. Shifting attention to repressors, we then found that competition between repressors and activators for binding sites is an insufficient mechanism of repression [29]. We found evidence in favor of a short range repression mechanism for two of the TFs, consolidating experimental evidence that exists for this mechanism. However, our results also raised the possibility that long-range mechanisms (such as direct interaction with the BTM) may also contribute to the repressors' function. We also studied the importance of cooperative DNA-binding (of both activators and repressors) in this system. Our results provide clear evidence of cooperative effects of some TFs but give mixed signals with respect to other TFs.

We also used our model to examine a contentious evolutionary issue. Several studies [30]–[32] have reported that TF binding sites undergo rapid turnover (loss and/or gain) during evolution. However, due to the difficulty of establishing true functionality of binding sites in practice (e.g., binding to a TF does not necessary lead to regulatory function [33]), it is not clear whether such turnover is largely limited to non-functional sites. We investigated this issue using our model in conjunction with evolutionary sequence comparison, and found that lineage–specific losses affect functional sites to a noticeable extent.

### Comparison to previous models

As mentioned above, a few thermodynamics-based models have been proposed in the past, which we now review briefly. The approach of Reinitz and colleagues exploits physicochemical principles, and includes important mechanistic aspects such as short range repression through quenching [13], [34]. However, the Reinitz model does not consider all possible molecular configurations, a fundamental tenet of the statistical thermodynamic treatment. Also, cooperative DNA-binding by TFs is not included in the model. Segal et al. [6] presented a model based on enumeration of all configurations of bound and unbound TFs. This model uses statistical thermodynamics to model TF-DNA interactions and to compute relative probabilities of configurations, but models the mapping from these configurations to their transcriptional output in a heuristic manner. Also, the Segal model ignores important mechanistic issues such as transcriptional synergy (discussed above) and short range repression. Furthermore, the formulation of transcriptional output in this model makes the computational task intractable. (The authors adopted sampling methods to deal with this issue, thereby sacrificing exactness of the model computation.) The models developed by other researchers make various simplifying assumptions, e.g., binding of a single activator is strong enough to activate transcription [14], and their implementations are often limited in their generality, e.g., only sequences with a small number of binding sites are considered [12], or all sites are assumed to have identical binding affinities [14]. See Table 1 for a summary of the strengths and weaknesses of the models discussed above.

**Table 1:** Thermodynamics-based models of gene expression and their properties.

Model	Enumeration of states	Variable site affinity	Cooperative DNA-binding	Transcriptional synergy	Short range repression
Shea- Ackers [5]	Y	N	Y	Y	N
Reinitz [34]	N	Y	N	Y	Y
Papatsenko [14]	Y	N	Y	N	Y
Segal [6]	Y	Y	Y	N	N
This paper	Y	Y	Y	Y	Y

‘Y’ = Yes, ‘N’ = No. “Variable site affinity” indicates whether the model implementation as described in the respective paper(s) allows the input sequence to have an arbitrary number of binding sites with variable affinities.

We have not undertaken a rigorous comparison of our approach versus the above-mentioned approaches, for three reasons. First, none of the previous models have a publicly available implementation that we could use in our setting. Bauer & Bailey's implementation [11] of the Reinitz model comes closest, but cannot be applied to more than one CRM at a time. Second, while Segal et al. [6] make their data set (and their predictions for this set) available, their method uses a much larger number of free parameters (the position weight matrices of the TFs were estimated from data), precluding a fair comparison. Third, and most importantly, our main goal in this study was to search for mechanistic insights that are revealed by the data, rather than engineering a model with the best possible fit to the data. For the same reason, we have not attempted here to position our work in comparison to machine learning-based models of gene expression [35], [36]. To facilitate future studies by other researchers, we make the source code of our implementation freely available online.

## Methods

The components of the thermodynamic system we consider are: (a) the DNA segment forming the regulatory sequence (CRM), (b) transcription factor (TF) molecules, and (c) the basal transcriptional machinery (BTM). A TF molecule may bind the CRM at any binding site (assumed of a fixed length), with site-specific affinity. The BTM may bind at the core promoter of the gene, and it initiates transcription when thus bound. We assume, following Shea & Ackers [5], that the level of gene expression depends primarily on the rate of transcription initiation.

### Statistical thermodynamics of gene expression

**Figure 1:**
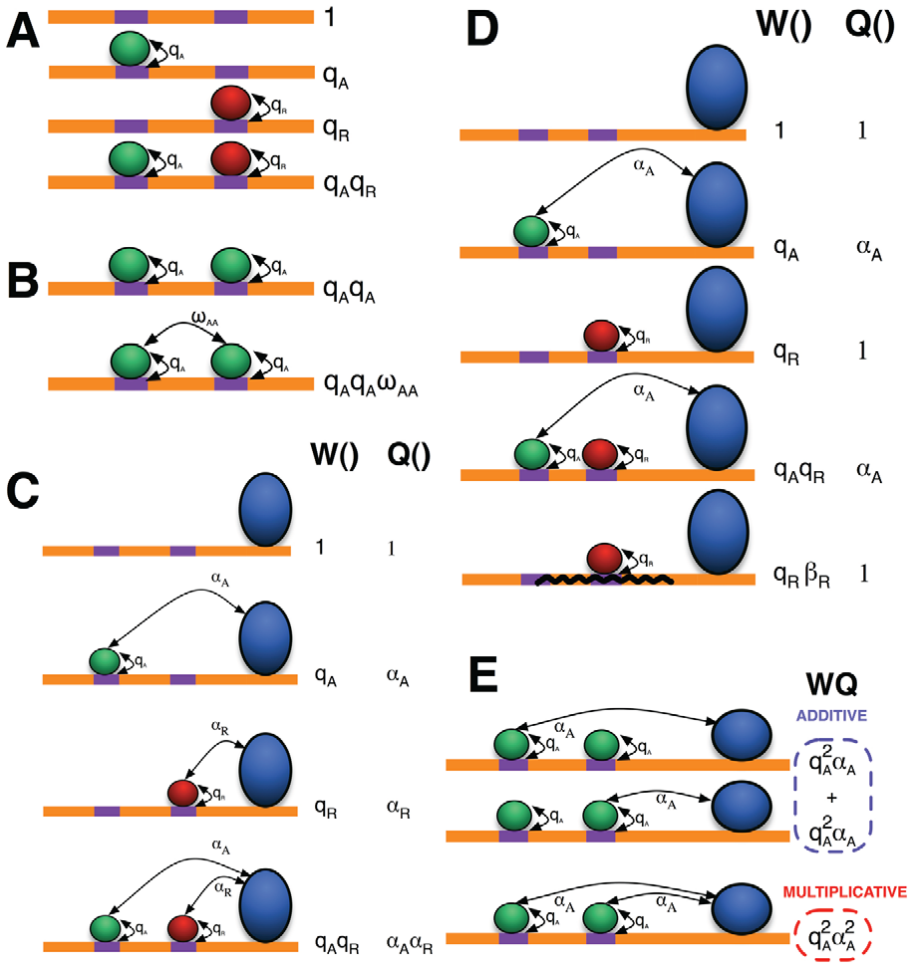
Statistical thermodynamic models of gene expression. (A) All possible molecular configurations of a CRM with two binding sites (purple), that may or may not be bound by a transcription factor (green circle = activator, red circle = repressor). The statistical weight *W* of each configuration is shown to its right. Each occupied site makes a contribution to *W* in a multiplicative fashion. (B) Cooperative DNA-binding is modeled by introducing a multiplicative factor (ω) to the statistical weight of a configuration. The same configuration is shown along with its statistical weight *W* under a model with no cooperativity (top) and a model with self-cooperative DNA-binding (bottom). (C) Statistical weight contributions from TF-DNA interactions (*W*) and from TF-BTM interactions (*Q*) for each configuration, in the Direct Interaction model (blue circle = BTM). Each bound activator or repressor molecule contributes to the TF-BTM interaction term (*Q*) in a multiplicative fashion. The statistical weight also receives a contribution from BTM binding at the promoter; this term is not shown here. (D) Same as (C), but for the short range repression model. A bound repressor (red circle) does not have a direct interaction with the BTM. Also, there is one additional configuration allowed here, as compared to Direct Interaction: one where repressor is bound and “effective” in shutting down its neighborhood for binding at activator sites (bottom). The statistical weight (W) of this configuration is scaled by a factor of β_R_, reflecting the strength of the repressor to change the chromatin accessibility. (E) Two ways to model the action of multiple bound activators: “additive effect” (top 2 configurations) and “multiplicative effect” (bottom). The total statistical weight (W×Q) under each model is shown. In the former, only one bound activator may contact the BTM in any configuration, while the latter has no such restriction and leads to transcriptional synergy.

We begin with an overview of the statistical thermodynamic theory of gene expression, following Buchler et al. [7]. This theory has two components, one dealing with the occupancy of TFs at DNA sequences, and the other with the interactions of occupied TFs with the BTM. We first describe the model of TF occupancy. Consider a CRM with *n* binding sites (e.g., *n = 2* in Figure 1A). A *molecular configuration*, denoted by σ, specifies which sites are bound and which are free. Thus there are *2^n^* possible configurations. The *statistical weight* of configuration σ, denoted by W(σ), and which we shall endeavor to compute, gives us the *relative probability*, P(σ), of the configuration when the system is in equilibrium. In other words, we have P(σ) = W(σ)/Z, where Z is a normalization constant, defined as ∑_σ_ W(σ), and known as the *partition function*. Calculation of P(σ) would allow us to answer questions like: “What is the relative probability of site *S* being in the bound state?” This may be computed by summing P(σ) over all σ in which *S* is bound, and is also called the *fractional occupancy* of the site *S*. The statistical weight W(σ) depends on the number and affinities of the occupied binding sites in the configuration σ, and on interactions between bound TF molecules. We will present details of W(σ) when discussing specific models below.

We next describe, at a high level, how the above molecular configurations (σ ) affect gene expression. We assume that the gene expression level (on a scale of 0 to 1) is equal to the fractional occupancy of the promoter by the BTM. Each of the configurations σ considered above (specifying bound or unbound TFs) may now correspond to two states, depending on whether BTM is bound or not. The statistical weight of these two states will be given by W(σ)Q(σ) and W(σ) respectively, where W(σ) is the contribution from TF–DNA interactions as explained above, and Q(σ) is the contribution from TF–BTM interactions, present only if the BTM is bound. Q(σ) depends on the bound TFs in the configuration σ, and may be construed as the transcriptional output from the configuration. We now calculate the relative probability of bound BTM as Z_ON_ = Σσ W(σ)Q(σ), and that of unbound BTM as Z_OFF_ = Σσ W(σ), to obtain the gene expression level as follows (note that “ON” and “OFF” represent the state of BTM occupancy, which is separate from the occupancy states of binding sites in the CRM sequence, as indicated by σ):
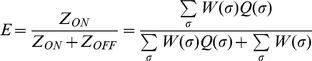
(1)


### Direct Interaction (DirectInt) model

Here, we present details of how the W(σ) and Q(σ) terms are specified by the first of our two models. Under this model, DNA-bound transcription factors interact favorably (activators) or unfavorably (repressors) with the BTM, thus affecting the probability of the BTM being bound at the promoter. We call this the Direct Interaction (“DirectInt”) model.

For a configuration σ, the statistical weight W(σ) has terms reflecting binding of TFs to their binding sites, and those reflecting interactions between TFs. Let *q(S)* denote the contribution of a single occupied site S to W(σ). This depends on the concentration of the TF and the strength of the site, and is given by:
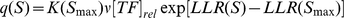
(2)where

- [TF]_rel_ is the concentration of the TF relative to some value ν,
- *LLR*(⋅) is the log likelihood ratio score of a site, computed based on the known position weight matrix (PWM) of the TF and the background nucleotide distribution [10],
- *S_max_* is the strongest binding site of the TF and *K(S_max_)* is its association constant.

(See Text S1 for how Equation (2) is derived.) Note that two unknown constants, one related to TF-DNA binding *(K(S_max_))*, and the other (ν) a constant of proportionality for TF concentration, appear together as a product, which can be treated as a single free parameter to be estimated from data. The above equation makes the implicit assumption that the binding energy of each position of a site is additive. This assumption has been questioned in several studies [37], [38], but is necessary in our case because there is not enough TF-DNA interaction data to construct accurate models incorporating higher-order interactions. Furthermore, the additivity assumption seems to be a reasonably good approximation for the TFs in the segmentation system [6], [13]. The statistical weight of a configuration σ, in the absence of cooperative binding, is then given by 
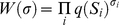
, where σ*_i_* is an indicator variable (values 0 or 1) for *S_i_* being occupied by its TF in the configuration (Figure 1A) [7].

If two bound TFs interact (protein–protein interaction), they make an additional contribution to the statistical weight of the configuration. We denote this contribution by ω(*d*), where *d* is the distance between their binding sites (Figure 1B). The dependence of this cooperativity term on the distance is discussed in Text S1. The statistical weight of a configuration, accounting for cooperative binding, is the product of contributions of all occupied sites and all TF-TF interactions implied by that configuration [7]:
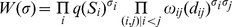
(3)where ω_ij_(*d_ij_*) denotes the statistical weight contribution due to interaction between the TFs bound to sites *S_i_* and *S_j_*, and *d_ij_* is the distance between these sites. We assume that cooperative binding is possible only if the bound sites are adjacent in the configuration, i.e., there is no other bound site in between. We also assume that it is predetermined whether any given pair of transcription factors exhibit cooperative binding or not. The model allows interactions between adjacent binding sites that may be either homotypic (of the same TF) or heterotypic (of different TFs).

Next, we describe how we model Q(σ), the statistical weight contribution from TF-BTM interactions. We assume that each TF is either an activator or repressor. A bound activator *A* interacts with the bound BTM with statistical weight *α_A_>1*, while a repressor *R* interacts with weight *α_R_<1* (Figure 1C). Q(σ) is the product of the *α* terms corresponding to each bound TF in the configuration. This corresponds to the intuition that a bound activator makes the configuration more energetically favorable (thus, a greater weight) while a bound repressor makes it less favorable. We also assume that each bound TF interacts independently with the BTM, with energy contributions that add up, which is reflected in the statistical weights being multiplicative.

Computation of Equation (1) involves summation of an exponential number of configurations. We developed an efficient algorithm based on dynamic programming to carry out the computation (see below and Text S1). We note that Gertz et al. made the same model assumptions [12], but, unlike their method, we provide a general and efficient implementation that is applicable to arbitrary sequences. The DirectInt model presented here largely follows Buchler et al. [7], with the treatment of sequence-specific DNA binding (Equation 2) being borrowed from Berg & von Hippel [9]. However, the approach of Buchler et al. [7], designed for prokaryotic systems, assumed repressors to work by competition with the polymerase, and does not extend to distally located binding sites.

### Short-range repression (SRR) model

In the DirectInt model above, repressor action is independent of the location of binding sites for repressors or activators. However, experimental work has shown that certain repressors act on activators only if they are bound within a “short range”, e.g., less than 150 bp, of the activator binding site [17]. Such short range repression, also called “quenching” [17], may work by repressors inhibiting DNA-binding of activators [39], possibly by modifying chromatin accessibility. We model this mechanism by assuming that a bound repressor does not directly interact with the BTM, instead, it creates a new possible configuration, one where DNA in its “neighborhood” (defined by a range parameter *d_R_*) is inaccessible to binding by any other TF, for example by localized chromatin modification (Figure 1D). A configuration where the neighboring chromatin is inaccessible (Figure 1D, bottom) competes with the configurations where the chromatin is accessible to activators, thus effectively reducing the occupancy of activators. We call this model the short-range repression, or SRR, model.

Note that there are more configurations under this model than in the DirectInt model. In any configuration, an activator site may exist in one of two states (bound or unbound) as in DirectInt. In contrast, each repressor site may now exist in one of three states: unbound, “bound-only”, and “bound-effective” (the bound-only state has the repressor bound but not interacting with either the BTM or the neighboring DNA, while in the bound-effective state the bound repressor makes the neighboring DNA inaccessible). Not all possible configurations are allowed, however. We assume that within a certain range of a bound-effective repressor, an activator site is not allowed to be bound (thus implementing the idea of short-range repression).

For a legitimate configuration σ, W(σ) in the SRR model is given by Equation (3), multiplied by a repressor-specific constant *β_R_* for each bound-effective site of the repressor *R* (Figure 1D, bottom). The parameter *β_R_* may be interpreted as the equilibrium constant of the reaction that changes the chromatin state from accessible to inaccessible. The value of *β_R_* controls the strength of the repressor. When it is close to 0, there is no repression effect; when it approaches +∞, the repressor completely shuts down all activator sites in the neighborhood. Thus, in this alternative to the DirectInt model, repression is modeled by augmenting the calculation of W(σ), instead of direct interaction terms (*α_R_*) for the repressor in Q(σ). Q(σ) is now a product of the direct interaction terms (*α_A_*) for activators alone. We show that even with this new model, it is possible to perform efficient computation of Equation (1) using dynamic programming (see Text S1).

### Modeling the action of multiple activators

We consider the following question: how are the effects of multiple bound activators combined? In both models described above (DirectInt, SRR), their individual statistical weights (*α_A_*) were multiplied, in calculating the overall contribution of activator-BTM interactions. This is the “multiplicative effect” model of combined action by multiple activators. It reflects a scenario where the bound activators interact with different parts of the BTM (or different steps of transcription initiation), and the energy terms are added. Veitia [26] shows that this multiplicative effect model results in “transcriptional synergy”, where the activating effect of two binding sites is greater than the sum of their individual effects, even in the absence of cooperative DNA-binding. We next consider an alternative scenario where in any given configuration, at most one activator molecule may interact with the BTM. This is plausible if for example the bound activators must interact with the same subunit of the BTM. In this case, the TF-BTM interaction term is written as Q(σ) = Σ*α_i_*, where the sum is over bound activators in the configuration. This is the called the “additive effect” model (Figure 1E). In this case, there will be no synergistic activation due to TF-BTM interaction, though some level of synergy may still arise from cooperative DNA-binding by activators. In Text S1, we compare the two mechanisms that may lead to transcriptional synergy: multiplicative effect model, and additive effect model in combination with cooperative DNA binding. The basic insight is that synergistic effect will disappear at high activator concentration under the cooperative binding model (activator binding has already been saturated under this condition, thus cooperative interactions will not be further helpful), but not under the multiplicative model. This difference in model behavior suggests that it is theoretically possible to distinguish two models from the data. To investigate the mechanism of synergistic activation, we implement both “multiplicative effect” and “additive effect” models as special cases of a more general model for combined activator action: a user-defined parameter *N_MA_* (positive integer) sets the limit on the maximum number of bound activators that can simultaneously interact with the BTM. We call this the “limited contact” model of activator action (see Text S1 for details). The cases *N_MA_* = 1 and *N_MA_* = ∞ correspond to the additive and multiplicative effect models respectively. This general model can be combined with cooperative binding of TF molecules, thus allowing us to study the relative contribution of multiplicative activation and cooperative binding.

### Algorithms for computing expression of a sequence

As discussed earlier, the computation of Equation (1) involves summation of an exponential number of configurations. In this section, we describe an efficient algorithm for computing the DirectInt model with multiplicative effect of activation. (The algorithms for other models are based on similar dynamic programming techniques and are presented in Text S1.) Let *Z_OFF_(i)* denote the total statistical weight of all configurations of sites up to the site *i*, with site *i* being occupied. We obtain the following recurrence, by summing over the position of the occupied site *j* nearest to site *i*:
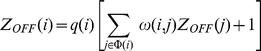
(4)where *q(i)* is the statistical weight of the site *i*, as defined in Equation (2), *ω(i, j)* is the interaction between the occupied sites *i* and *j*, and *Φ(i)* is the set of sites to the left of *i* that do not overlap with *i*. This recurrence equation is similar to that in [40], [41]. The constant term, +1, corresponds to the case where no site to the left of *i* is occupied. Under this model, *Q(σ)* is the product of the transcriptional effects (α terms, as described before) of all occupied TF molecules in the configuration *σ*. Let *f(i)* be the factor bound at the site *i*, and *α_f(i)_* be the transcriptional effect of *f(i)*, then we have the following recurrence for Z_ON_:
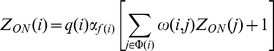
(5)To calculate the values required for Equation (1), we simply take the sum over all possible values of *i*: 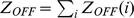 and 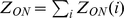. The time complexity of the algorithm is *O(n^2^)*, where *n* is the number of sites in the sequence. However, if cooperative interaction between adjacent sites is not modeled, or the interaction only occurs within a constant range, the time complexity is linear in *n*.

### Data

We started with the *Drosophila* segmentation data set from Segal et al. [6]. This set includes 44 *bona fide* CRMs with their A/P expression profiles, eight TFs (*Bcd*, *Cad*, *TorRE*, *Hb*, *Gt*, *Kni*, *Kr*, *and Tll*) with their concentration profiles and PWM motifs. Each expression profile (or concentration profile) consists of 100 real numbers between 0 and 1 representing the relative expression level of the CRM (or relative concentration of a TF) in positions along the A/P axis, divided into 100 bins from anterior to posterior. One problem with this data set is that not all relevant TFs in the terminal regions (e.g., *Slp1*) are included or known [42]. Also, the *TorRE (Torso Response Element)* motif included in this data set is assumed to correspond to a (yet unknown) TF that has activating role in the terminal regions of the embryo. Recent evidence suggests that on the contrary *TorRE* may correspond to the *Capicua* transcription factor, which is a repressor expressed in the trunk region of the embryo and post-transcriptionally degraded at the termini in response to Torso signaling [43]. This casts doubts over the inclusion of *TorRE* and in general the terminal regions of the expression profiles as part of the data set, especially for evaluating models that distinguish between activator and repressor mechanisms. We thus limited the CRM expression profiles to their portions lying between 20% and 80% egg length. The number of CRMs came down to 37, after excluding those without patterned expression in this spatial range. This final data set included six motifs (*Tll* and *TorRE* were excluded), of which five (*Cad*, *Gt*, *Hb*, *Kr*, *Kni*) were taken from Noyes et al. [44] and one (*Bcd*) was obtained from FlyREG [45]. Binding sites were annotated as those with log likelihood ratio (LLR) scores greater than 0.4 times the LLR score of the optimal site [46]. This threshold is weak enough to include a large number of putative sites for each TF, while keeping the running time low.

### Model training

Parameter training was performed using the Nelder-Mead simplex method and the quasi-Newton method, and restarts were used to deal with potential local optima. Optimization of the correlation coefficient between predicted and known expression values was alternated with optimization of the sum of squared errors (See Text S1 for details). Note that model training is performed separately for each model (DirectInt or SRR, with or without cooperative interaction, etc.). Thus, even though two models may share certain parameters, e.g., (*K(S_max_*)ν), their values may be different under the two models after training. The running time of the program scales linearly with the number of TFs, and the total length of sequences (for all models except the “limited contact model”, see Text S1). In our dataset, with 6–8 TFs and about 40 CRMs of average length 1450 bp, the parameter training phase took about 3–4 hours of running time on a desktop computer with 2.2GHz CPU and 2GB memory.

## Results

Here we present “GEMSTAT” (Gene Expression Modeling based on Statistical Thermodynamics), an efficient and publicly available implementation of models for predicting expression from sequences, given TF concentration profiles and TF binding motifs (PWMs). GEMSTAT can be trained on any number of CRMs with known expression profiles. It can be easily configured to use one of many possible combinations of mechanistic features of a rigorous thermodynamics-based model of promoter occupancy. Details of the model are provided in Methods. Here, we begin with a brief summary of the implemented features, and use GEMSTAT to gain insights into mechanisms of transcriptional regulation in the *Drosophila* segmentation network.

### Models and evaluation

GEMSTAT offers the following choices between various model features:

- Direct Interaction (“DirectInt”) model or Short Range Repression (“SRR”) model. Both prescribe direct interactions between bound activators and the BTM, and differ in how repressor action is modeled. In the DirectInt model, bound repressors have direct, destabilizing interactions with the BTM, while in the SRR model they function by rendering the neighboring chromatin inaccessible.
- Additive or multiplicative model of activator action. These differ in how the effects of multiple bound activators are combined. The multiplicative model allows any number of activators to simultaneously interact with the BTM, leading to synergistic activation of transcription (“transcriptional synergy” [8]), and the additive model allows only one such interaction in any configuration. These two models are in fact special cases of a more general framework, called the “limited contact model”, by which any desired limit may be imposed on the number of simultaneous activator-BTM interactions, and thus on the extent of transcriptional synergy among activator sites.
- Cooperative DNA binding. If this option is chosen for a pair of TFs, two molecules bound at “adjacent” sites (i.e., a pair of sites with no other occupied site in between) are assumed to interact favorably, thus exhibiting cooperative DNA binding. We support both homotypic and heterotypic interactions between TFs.

The above choices are accompanied by parameters that may be set manually, and some of which may be left as free parameters to be trained from the data. All model parameters are described in Table S1. The program takes as input the sequence and expression profiles of a set of CRMs, and the PWMs and concentration profiles of a set of TFs. Expression profiles and concentration profiles are specified as vectors of a fixed dimension, allowing it to be easily used to model any regulatory system. (In our application, vector components correspond to positions along the A/P axis of the embryo, but in other applications these could be distinct anatomical domains or temporal points.) The source code is available at http://veda.cs.uiuc.edu/Seq2Expr/.

The data set consists of 37 experimentally characterized CRMs driving patterned expression along the anterior-posterior axis of the blastoderm stage *Drosophila* embryo (see Methods). We used several different approaches to objectively evaluate a model and compare models. Our first metric of model performance is the correlation between the model predictions and the observations. For each CRM, we calculated the Pearson correlation coefficient (CC) between the predicted and the observed expression profiles (over 60 bins), and computed the average CC over all CRMs. We also recorded the number of CRMs with CC>0.65. Additionally, we estimated for each CRM the significance of improvement (in CC) due to one model versus another, and combined these estimates into a *p*-value of improvement over the entire data set, as described in Text S1. We also calculated the average CC under 10-fold cross validation (denoted by “CVCC”), as a test of predictive ability, and for fair performance comparison between models with different numbers of parameters. For any given choice of model, an identical model that uses randomly permuted PWMs was evaluated as negative control. Any observation about model comparison based on correlation coefficients was also confirmed by visual inspection of the predicted expression patterns on all 37 CRMs. We note that there is no consensus yet on the most reasonable way to evaluate predictions of expression models for data sets such as that used here. We chose the correlation coefficient because of its ability to capture the salient pattern along the A/P axis, and we based all of our claims on this measure to keep our analysis objective and unbiased.

### Cooperative DNA-binding by transcription factors

**Figure 2:**
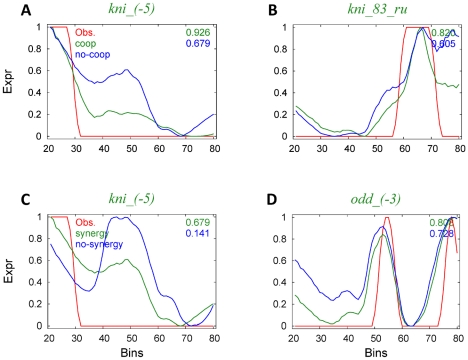
Effect of cooperative DNA-binding of TFs and the mode of transcriptional activation (multiplicative or not) on model performance. (A,B) Predicted expression profiles of a DirectInt model with no cooperativity (“no-coop”, blue) and a model with self-cooperative binding for *Bcd* and *Kni* (“coop”, green) are shown for each CRM, with reference to the CRM's known readout (“Obs.”, red). The correlation coefficient between a model's prediction and the known readout is indicated in the top right corner of the panel. Each expression profile is on a scale of 0 to 1 (scaling does not affect correlation coefficient), and shown for bins 20 to 80 (i.e., 20% to 80% egg length) of the embryo. Shown are two CRMs for which one model was deemed better than the other (CC≥0.65, difference in CC≥0.05). (C,D) Predicted expression profiles of a DirectInt model with multiplicative activation (“synergy”, green) and one with additive activation (“no-synergy”, blue). Shown are two CRMs where the multiplicative model is better than the additive model (CC≥0.65, difference in CC≥0.05). Self-cooperative DNA-binding was not used in this evaluation.

We began by exploring the effect of cooperative DNA-binding by molecules of the same TF, i.e., homotypic interactions. (Modeling heterotypic interactions would involve many more free parameters and was not pursued in this study). Segal et al. [6] also studied this effect, but since their model lacks mechanistic details of activation, the effect of cooperative binding may not be distinguishable from simultaneous interaction of TFs with the BTM (the “multiplicative effect”, also called “transcriptional synergy” [8]). As a baseline, we evaluated the DirectInt model that excludes any cooperative binding terms, but allows for transcriptional synergy. The average correlation coefficient (CC) of this model (of 13 free parameters) is 0.547, with accurate predicted readout (CC>0.65) on 16 of the 37 CRMs (Table 2). In contrast, 25 independent negative controls yielded a mean average CC of 0.211 (standard deviation of this mean across the 25 trials was 0.075). The cross validation correlation coefficient (CVCC) supports the high predictive ability of the model (average CVCC of 0.4, compared to 0.02±0.083 from negative controls). We then included self-cooperativity of each TF separately (only one additional parameter at a time), and computed the average CC and CVCC as before. Each of the TFs showed an improved CVCC over the baseline of no cooperativity across almost every replicate of the cross validation exercise (Table 2, Table S2), while *Bcd* and *Kni* showed the most pronounced effects of cooperativity in terms of average CC. When both *Bcd* and *Kni* were included as cooperative factors, the average CC improved further over the model with each factor alone. The improvement in going from no cooperativity (average CC = 0.547) to self-cooperativity for *Bcd* and *Kni* (average CC = 0.587) was highly significant (*p*-value 1.3E-6). Examination of the expression predictions on individual CRMs identified 12 CRMs where the cooperativity model was better and two where it was worse. (Two cases are shown in Figure 2 (A,B), and the complete list is in Figure S2.) Our results are consistent with Segal et al. [6], who found self-cooperativity to improve prediction. Moreover, we find this to be the case even in the presence of transcriptional synergy, which if not accounted for could have confounded the effects of cooperative DNA-binding by activators.

**Table 2:** Evaluation of the DirectInt model with various cooperativity parameters.

Model	# Pars	Avg. CC	#(CC>0.65)	CVCC (STDEV)
No Coop	13	0.547	16	0.400 (0.02)
Neg Ctrl No Coop	13	0.211±0.076	7.76±1.6	0.02±0.083
*Bcd* Coop	14	0.577	22	0.428 (0.01)
*Cad* Coop	14	0.553	21	0.428 (0.02)
*Gt* Coop	14	0.557	22	0.428 (0.03)
*Hb* Coop	14	0.552	20	0.328* (0.02)
*Kni* Coop	14	0.565	20	0.458 (0.02)
*Kr* Coop	14	0.550	16	0.441 (0.02)
All TF Coop	19	0.603	25	0.418 (0.03)
*Bcd* & *Kni* Coop	15	0.587	24	0.460 (0.02)
Neg Ctrl *Bcd* & *Kni* Coop	15	0.214±0.08	8.04±1.86	0.027±0.077

The models examined include those without self-cooperative DNA binding (“No Coop”), with cooperative binding by one of six different TFs (“*Bcd* Coop”, “*Cad* Coop”, etc.), with cooperative binding by all six TFs (“All TF Coop”), and with cooperative binding by *Bcd* as well as *Kni* (“*Bcd*&*Kni* Coop”). For each model, the number of free parameters used is shown (“# Pars”), along with average correlation coefficient (“Avg. CC”) between model prediction and true readout over all 37 CRMs in the data set, the number of CRMs where the average CC was above 0.65 (“#(CC>0.65)”), and the average correlation coefficient under a 10-fold cross-validation scheme (“CVCC”). Also shown are evaluation results for negative controls (“Neg Ctrl”) corresponding to the “No Coop” model and the “*Bcd*&*Kni* Coop” model. A negative control involved re-training a model with randomly permuted PWMs; shown are the average and standard deviation (of each evaluation metric) over 25 independent replicates of such a control.

*Note that the CVCC values depend upon how the data set got partitioned in the cross-validation exercise. The values in the last column are from one such partition (same across all rows). The *Hb* Coop model shows lower CVCC than the “No Coop” model in this partition. CVCC values from 6 additional partitions are shown in Table S2, and the *Hb* Coop model performs better than the No Coop model in five of those six cross-validation exercises.

**Figure 3:**
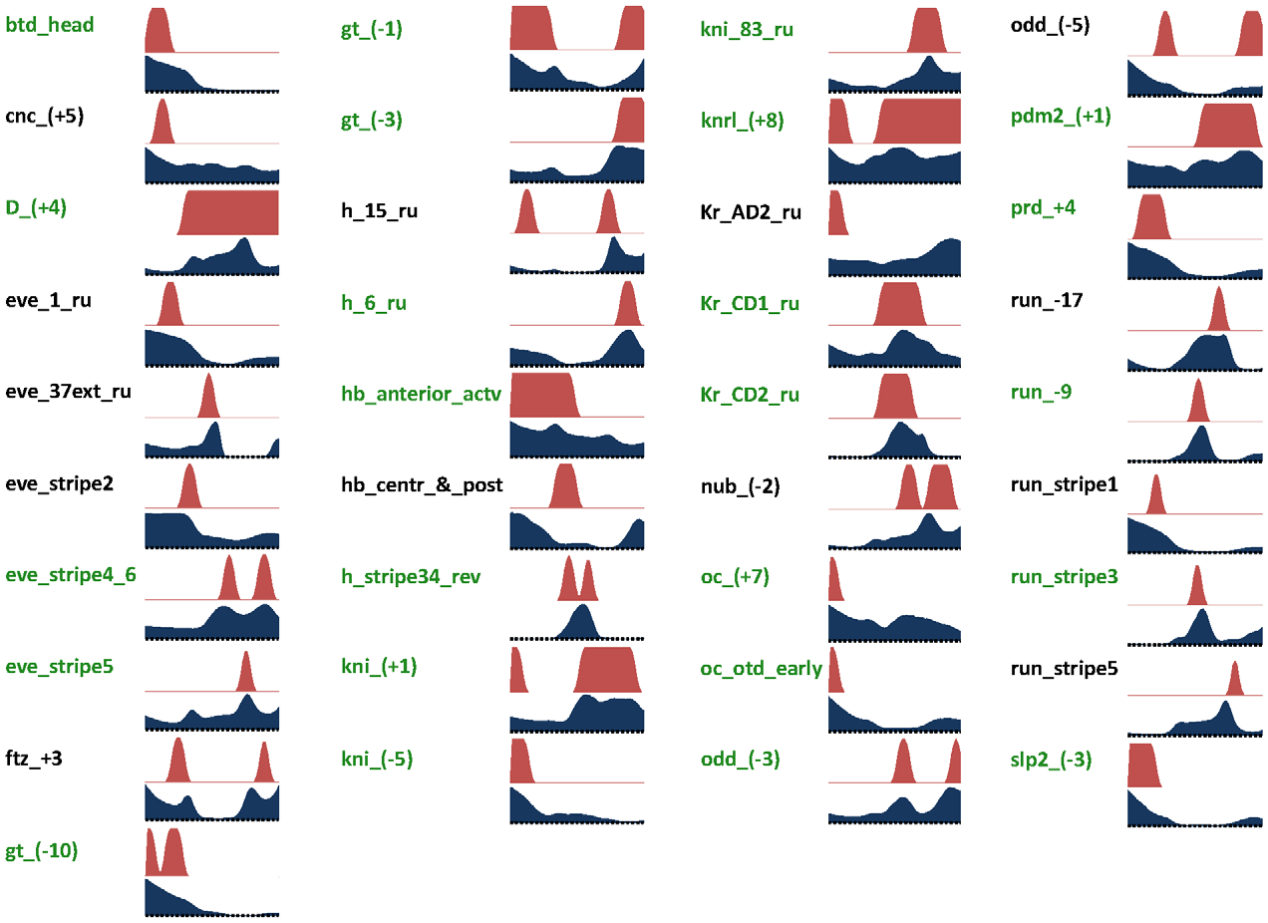
Model predictions. The predicted expression profile of the DirectInt model (with *Bcd* and *Kni* self-cooperativity) is shown (blue) in comparison to the known readout (red), for all CRMs in the data set. Each expression profile is on a scale of 0 to 1 (y-axis), and shown for bins 20 (left) to 80 (right) of the embryo. Labels in green indicate CRMs where the CC is greater than 0.65.

As a visual aid for interpreting the quantitative evaluations reported above, we present in Figure 3 all of the expression predictions from the above model (with *Bcd* and *Kni* self-cooperativity), alongside their respective known expression patterns. A detailed summary of the model's performance is given in Table 2, along with results from an appropriate negative control. This model was also fit to the entire data set of Segal et al. (44 CRMs, inclusive of terminal bins) and found to have slightly (but not significantly) higher average CC than the published predictions of the Segal model [6], although our model uses fewer free parameters (see Figure S3 for details.)

### Synergistic activation through simultaneous interaction of multiple activators with BTM

GEMSTAT implements two alternative approaches to combining the effects of multiple activator sites, using the parameter *N_MA_* described in Table S1: the additive effect (*N_MA_* = *1*) and the multiplicative effect (*N_MA_* = ∞), as well as approaches that are in between these two extremes. The “multiplicative effect” model allows any number of activator molecules to simultaneously interact with the BTM, which as discussed in Methods, leads to transcriptional synergy, a source of synergistic activation that is distinct from cooperative DNA-binding [8], [26]. We used the two extreme values of *N_MA_* to test whether this phenomenon leads to improved agreement with the data, while keeping other aspects of the model fixed (Table 3). The baseline model here was one with *N_MA_* = 1 (no synergy) and with no self-cooperative DNA-binding. The average CC from this model (0.516) improved significantly (to 0.547; *p*-value 3.7E-4) when we introduced synergy due to the multiplicative effect of multiple activators (*N_MA_ = ∞*). (This change does not involve any additional free parameters.) This was further confirmed by a greatly improved CVCC (0.295 to 0.40, see Table 3 and Table S3), as well as by examination of predictions for individual CRMs (Figure 2(C,D), and for the complete results see Figure S4): the multiplicative effect model showed clear improvements on 6 CRMs and was worse on 3 CRMs. These observations suggest that simultaneous interaction of multiple activators with the BTM is a plausible source of synergistic activation.

**Table 3:** Effect of transcriptional synergy on model performance.

Synergy	Cooperativity	Avg. CC	CVCC (STDEV)
N	N	0.516	0.295 (0.02)
Y	N	0.547	0.400 (0.02)
N	Y	0.558	0.292 (0.02)
Y	Y	0.581	0.396 (0.03)

A DirectInt model with or without transcriptional synergy (“Synergy = N(o)” versus “Synergy = Y(es)”) was evaluated by the average correlation coefficient (“Avg. CC”) on the 37 CRMs in the data set, as well as the average CC under 10-fold cross validation (“CVCC”). “Synergy = No” is implemented by setting *N_MA_* = 1 for the two activators (*Bcd* and *Cad*), while “Synergy = Yes” amounts to setting *N_MA_* = ∞. The evaluation is done in the presence of *Bcd* and *Cad* self-cooperative binding (“Cooperativity = Y”) as well as in the absence of any DNA-binding cooperativity (“Cooperativity = N”).

Cooperative binding was kept out of the model in the above test. We next introduced cooperative binding (only for the two activators) into the model, and examined the contribution of the multiplicative effect. We found that the model with both sources of synergistic activation shows better quality of fits compared to the model with cooperative binding alone, in terms of average CC (from 0.558 to 0.581, *p*-value 7.3E-11, see Table 3) as well as CVCC (0.292 to 0.396). We also confirmed this improvement by examination of individual CRMs: the model using multiplicative effect along with cooperative binding led to better fits for 8 CRMs compared to the model with cooperative binding alone (Figure S5) and was worse in no case. This result suggests that synergistic activation due to multiplicative effect of activators may be over and beyond that due to cooperative binding [26].

### Short range repression as a mechanism of repressor function

In all of the above tests, we had used a “Direct Interaction” model of repressor function, where a bound repressor is assumed to interact directly with the BTM, destabilizing the configuration, and thus curbing the roles of activator sites in the entire CRM. GEMSTAT also allows us to deploy a more “localized” form of repressor action, in the form of the short range repression (SRR) model, where a bound repressor makes the neighboring chromatin (within some range *d_R_*) inaccessible. Prior experimental work [20] suggests that the four repressors in our data set – *Kr*, *Hb*, *Kni*, and *Gt* – act over short distances (∼100–150 bp [14]), and in two of these cases (*Kr* and *Kni*) repression depends on the histone deacetylase dCtBP, which suggests a possible mechanistic basis for the short range action [20]. In our tests, we sought to examine if the SRR model implemented in GEMSTAT is realistic enough to capture the repressors' contributions to expression patterns.

**Figure 4:**
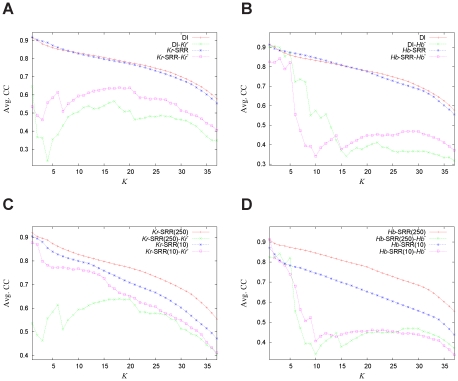
Evaluation of short range repression (SRR) model. (A,B) Two of the four repressors (*Kr and Hb*) are evaluated separately, by comparing predictions from a model where one repressor is modeled through DirectInt (“DI”) to predictions from a model where that repressor acts through SRR (“*Kr-*SRR”and “*Hb-*SRR”, in panels A and B respectively). For each model, the average correlation coefficient (CC) of the *K* best predictions (CRMs) of that model is shown, as a function of *K*. Also shown for each model is the average CC (over the same *K* CRMs) when the repressor is “knocked down” (e.g., “DI-*Kr^−^*”, “*Kr-*SRR-*Kr^−^*” in panel A). (C,D) The SRR model for (C) *Kr* and (D) *Hb* (with range of repression *d_R_* = 250 bp) is compared to the corresponding model at *d_R_* = 10 bp, where the repressor can only affect overlapping or adjacent sites. Semantics of the plots are as in (A–B).

Starting with a baseline where every repressor was modeled by “Direct Interaction”, we introduced the SRR model for one repressor at a time (with *d_R_* = 250bp), and compared the resulting model with the baseline. Although none of the four resulting models (*Kr-*, *Hb-*, *Kni-*, *Gt-*SRR) showed clear improvement over the baseline, we found strong evidence that for *Kr* and *Hb*, the SRR model implemented by GEMSTAT was able to capture the repressive effects of the factors almost to the same extent as the Direct Interaction model, as described next. We first extended our evaluation metric, the average CC, in the following way: we considered the best *K* CRMs for a model (in terms of CC), and plotted the average CC over these *K* CRMs, for all values of *K* (1 … 37). We found the *Kr*-SRR model to be highly similar (in terms of average CC) to the baseline model throughout the range (Figure 4A). Additionally, for each model and each value of *K*, we plotted the average CC of the same model under a *Kr* “knock down” condition, i.e., where the *Kr* concentration was set to 0 across the A/P axis. Such a “knock down” plot allows us to visualize the contribution of a TF (*Kr* in this case) to the model. We found *Kr* to contribute significantly to both models, although the contribution to the SRR model was not as strong as to the DirectInt model. This may reflect certain limitations of the SRR implementation in GEMSTAT, but the results strongly suggest that the short range nature of *Kr* action [20] is largely captured by our model. We also examined the performance differences between the models on individual CRMs. We found seven CRMs where the SRR model was as effective as or better than the Direct Interaction model in predicting readout, with a significant contribution from *Kr* (Figure S7). In five other cases, the Direct Interaction model yielded superior fits (plots not shown). Similar evidence for the effectiveness of the *Hb*-SRR model is shown in Figures 4B and Figure S8. However, the *Gt*-SRR model does not seem to elicit significant contribution from *Gt*, even though this repressor is found to be effective within the DirectInt model (Figure S6A). A similar lack of evidence was encountered for the *Kni*-SRR model (Figure S6B).

Thus, we find that for *Kr* and *Hb*, quenching of activator sites within a distance is sufficient to capture the repressive effect of the TFs, supporting the hypothesis that these two TFs act mainly as short-range repressors, confirming what has been reported in earlier studies, which admittedly relied on a small number of CRMs and synthetic enhancers. On the other hand, we did not find strong evidence of short-range repression for *Gt* and *Kni*, and even for *Kr* and *Hb* the SRR model's performance was only as good as and not better than the DirectInt model. This is somewhat unexpected; it may be in part due to limitations of our SRR model, but may also be hinting that these TFs use long-range repression mechanisms as well (see Discussion).

### Competitive binding is insufficient as a mechanistic explanation of repressor action

Repression by competitive binding, as proposed in the literature [7], [29], involves the binding of repressors to sites overlapping activator sites, thereby suppressing their occupancy by activators. This mechanism may be thought of as a special case of the SRR model in GEMSTAT, with the repression range parameter (*d_R_*) set to ∼10 bp. At such a small value of *d_R_*, a repressor can only make its immediate neighborhood inaccessible, equivalent to inactivation of overlapping activator sites. Having observed above that the *Kr* and *Hb* repressors are effectively modeled in the SRR mode, we compared the *Kr*-SRR and *Hb*-SRR models at *d_R_* = 250 to their respective versions at *d_R_* = 10. As shown in Figure 4 (C,D), in both cases the competitive binding model (*d_R_* = 10) was significantly worse than the SRR model, both in terms of average CC and in terms of the repressor's contribution.

### Evidence for functional contribution of lineage-specific sites

Finally, we sought to use the GEMSTAT program to probe an important question regarding the function and evolution of transcription factor binding sites. A number of recent studies have reported the “turnover” (evolutionary gain and loss) of binding sites, based on sequence comparison [30], [31], [47] or from ChIP-based experiments [48]. However, it is possible that such lineage-specific loss and gain are largely limited to non-functional sites, i.e., the false positive matches to PWMs, or sites that are bound by TFs but do not regulate expression [33], [49], [50]. Here, we explored this possibility by asking if sites that change in lineage-specific ways are functional in contributing to the expression patterns. We note that lineage-specific losses may in part be artifacts of alignment errors (i.e., sites were completely conserved but not deemed so, due to misalignment). However, in practice, the true gain/loss of sites may be hard to distinguish from alignment errors, so we will call both cases as lineage-specific changes here.

**Figure 5:**
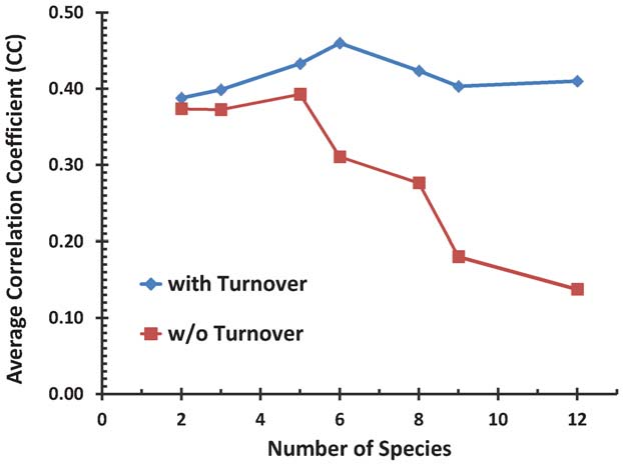
Effect of evolutionary filter on binding sites used in model. The average CC over all 37 CRMs of the DirectInt model (without cooperative DNA-binding) is shown. The x-axis indicates the number of species in which conservation of a binding site was required for it to be included in the model's input. The red curve corresponds to the case where the conservation filter does not allow turnover, i.e., the sites used in the model must be fully conserved across all species considered. The blue curve represents a conservation filter that allows turnover, i.e., where a site may undergo lineage-specific changes. Thus for “number of species” = 6, a site used in the model may be conserved in six or fewer species, as long as the conservation is deemed significant by the procedure described in Text S1.

We predicted sites by demanding that any predicted site be conserved (in the sense of being above threshold) in all species analyzed, and examined how the quality of fit varies as this evolutionary filter was made more stringent by including more species. We found that more conservative evolutionary filters lead to greatly reduced average CC (Figure 5, red). This shows that a noticeable part of the CRMs' functionality is carried by sites (in *D. melanogaster*) that are not found to be conserved across all phyla. Those sites could, broadly speaking (a) be deeply conserved in the examined phylogeny, but with some lineage specific losses or (b) have arisen specifically in *D. melanogaster* or a recent ancestor. Next, we modified the evolutionary filter to demand deep (but not necessarily complete) conservation across the phylogeny (see Text S1) and found that above-mentioned loss in quality of fits disappears (Figure 5, blue, compare number of species = 2 vs. 6). Since the new evolutionary filter discards sites of type (b) mentioned above, we inferred that a noticeable part of the CRMs' functionality is carried by sites that are largely conserved but also undergo lineage-specific losses.

## Discussion

One of the grand challenges in biology today is to understand how the control of gene expression patterns is encoded in the DNA. The common response to this challenge has been to identify individual regulatory interactions (between transcription factors and genes) that are necessary for the proper expression pattern, and correlate or attribute such interactions to the presence of binding sites near the gene. In order to determine if the knowledge of regulatory interactions gathered in this way is accurate and complete, we need a test of its predictive ability. In regulatory systems such as those involved in metazoan development, where regulatory output is a complex function of strong and weak binding sites and varying levels of transcription factor presence, such tests require a quantitative model that maps a regulatory sequence to its expression readout, based on input information about transcription factor levels in the cell. Failures of the model to explain available data can guide us to missing pieces of the regulatory system or potential flaws in our understanding of how inputs relate to outputs. We have developed statistical thermodynamics-based models of gene expression that can be valuable in such an enquiry. We used these models to study a number of mechanistic issues including the action of repressors, the combined effect of multiple activators, and cooperative DNA-binding by transcription factors, as well as the evolutionary dynamics of binding sites. Our results provide novel insights as well as support for existing hypotheses.

In contrast with earlier mechanistic enquiries [3], [4], [51]–[53], which were based on experimental analysis of a small number of genes or synthetic regulatory sequences, our analysis is based on a large set of CRMs and expression patterns. In the future, this may emerge as a new paradigm for mechanistic explorations of transcriptional regulation (“the regulatory code”), especially with the availability of higher resolution expression data. In addition to finding evidence for specific mechanistic hypotheses, our approach may be used to suggest specific experiments to test such hypothesis. For example, once a CRM is found to have widely different predictions under two alternative models, suitable biochemical and/or genetic experiments may be designed to demonstrate the underlying mechanism. Our model, being based on general physicochemical principles and having flexible schemes of modeling different aspects of transcription, has broad applicability regardless of the specific regulatory system being studied. For the same reason, if its application to a particular system reveals disagreements with data, it can alert us to the possibility of missing components or mechanisms. We make the software used in training and testing our models freely available, in the hope that this will facilitate its broader application to other systems.

### Findings and limitations

Three different mechanisms have been previously hypothesized for repressor action: (i) competition with activators for access to binding sites, (ii) direct interference with BTM recruitment and assembly, and (iii) local interference (“quenching”) with the function of nearby activator sites. The last hypothesis seems to be most likely in the context of the regulatory system we analyze, as suggested by the following observations: first, repressor and activator sites are often found to be close to each other [19]; second, CRMs of the same gene often work independently, i.e., a repressor site within one module does not stop the function of another module for the same gene [54]; third, some repressors are found to depend on a co-repressor, CtBP, a histone deacetylase that presumably increases the association of nucleosomes to DNA, making it less accessible [20]. However, direct evidence of this so-called short-range repression (SRR) phenomenon is limited to a few CRMs and synthetic enhancers [20]. We implemented models that could investigate all three mechanisms with respect to their agreement with data on a moderate number of CRMs. Note that even though the short range mechanism has been implemented (in other forms) previously [13], [14], it has never been tested within a framework that also implements alternative mechanisms. We report the first direct data-based comparison between alternative hypotheses regarding repression. Our results clearly exclude the hypothesis of competitive binding being the main mechanism of repression, and are consistent with the SRR hypothesis for two of the four repressors studied (*Kr* and *Hb*). It is somewhat unexpected that the SRR model does not explain the data as well as the DirectInt model for *Gt* and *Kni*. We note that while *Gt* is believed to be a short range repressor, Nibu et al. [20] leave open the possibility of this protein having long range mechanisms of action as well, in light of the fact that it does not require dCtBP to mediate repression. Similarly, *Kr* has been found to have long range mechanisms as well [21], [55]. It is also likely that to some extent the inability of the SRR model to match (for *Gt* and *Kni*) or exceed (for *Kr* and *Hb*) the effectiveness of the DirectInt model arises from shortcomings of our model and evaluation procedure. Our model assumes that once a repressor molecule is bound, it will make its entire neighborhood inaccessible, defined by a range parameter. We would intuitively expect that the repression effect is stronger for closer chromatin regions, and this is not modeled due to our lack of understanding of the exact mechanism by which repressors may change the chromatin structure. Similarly, we do not know exactly how the effects of two repressor molecules are combined in the regions that may be affected by both, and this part is treated in a simplistic manner under our SRR model. The dataset may also limit our ability to study detailed mechanisms: the resolution of expression patterns is low and the dataset lacks informative negative controls (all sequences are wild type CRMs). Finally, our tests are likely to have been weakened by the fact that models are compared on individual CRMs and not entire control regions. It is generally assumed that the short range mode of repressor action is necessary for the functional modularity of CRMs. For example, *Kr* is a key input to the *eve stripe 2* enhancer, but it can adversely affect the expression readout of the adjacent *eve stripe 3* enhancer; this interference is avoided presumably because of its short range of action [56], [57]. Thus, the effect of SRR is already manifested in the compactness of CRMs, and if it were possible to compare SRR with the direct interaction model on entire gene control regions, we would likely observe a clear advantage to the former. Despite these limitations, the SRR model along with a detailed activation model allows to ask questions that cannot be addressed with simple non-mechanistic models of CRM function.

Another important issue we explored is how multiple activator sites contribute to expression. It is likely that this multiplicity is important for the synergistic activation, where the total effect of multiple sites is larger than the sum of their individual effects. That such synergy is real and important has been shown through *in vitro* experiments on the effect of the number of sites [51], as well as *in vivo* experiments on the typically sharp boundaries of gap gene expression domains [2]. Mechanistically, synergy may result either from cooperative DNA binding of multiple activator molecules or from simultaneous interaction of multiple activators with the BTM (Text S1) [26]. Our model implements both mechanisms, and is thus able to examine the effect of each mechanism on readout, both in the absence and in presence of the other mechanism. We found that both mechanisms are involved in setting the precise expression profile; the effect of transcriptional synergy is evident, and complementary to that of cooperative binding. We have not explored in this study some important details on how synergistic interactions with BTM may occur, and these may worth further investigation. For example, we did not make any distinction between different activators. It is plausible that two different activators may interact with BTM simultaneously, contacting different subunits [52], while the two molecules of the same TF may act in an additive fashion, contacting the same subunit. Other possibilities remain to be explored with regard to cooperative DNA-binding as well. One possibility stems from our assumption that only two adjacent bound molecules may interact with each other. Although this assumption has been commonly made in other studies dealing with cooperativity [6], it is based partly on computational considerations and partly on our lack of understanding of the mechanistic details of interactions among TF molecules. On the topic of mechanistic limitations of our models, we note also that in equating gene expression to the fractional occupancy by the BTM, we are ignoring the internal dynamics of transcription initiation and elongation [58]–[60].

We found that for a number of CRMs, the model (mis-)predicts expression outside the CRM's primary expression domain(s). For instance, the CRM “kni_(-5)” drives anterior expression only, but the model additionally predicts modest expression in the central and posterior regions of the embryo (Figure 3). We noted that kni_(-5) has many binding sites for *Cad*, which is an activator present in the posterior half of the embryo. Presumably, the model fails to find strong evidence of appropriate repressive influence, and predicts kni_(-5) to drive expression in the posterior regions, mediated by the putative Cad sites. A similar observation was made with respect to the CRM “eve_stripe5”, which drives expression in the posterior half (in a stripe between bins 60 and 70, see Figure 3). This CRM harbors several high quality putative sites for Bcd, which is an anterior activator, and this is presumably the reason why the model predicts modest anterior expression as well. That such incongruous predicted expression is often seen under multiple models suggests that the errors may not be due to the specifics of the model that we have been varying. Rather, it is possible that we are missing some additional repression mechanism, e.g., from chromatin modifications, from unknown repressor sites, or mischaracterization of binding affinity. A relevant fact worth noting here is that there is some ambiguity about the appropriate binding profile to use for the important repressor Gt. In the current study, we used the profile estimated from in vitro Bacterial-one-hybrid (B1H) experiments [44], which happens to be quite different from the profile estimated from verified Gt binding sites in DNA footprinting experiments [45]. However, because relatively few sites were verified, the footprinting-based Gt profile is too un-specific to be used for prediction of new sites. We observed that the total number of Gt sites in all CRMs is considerably smaller than most other factors. This may have led to underestimation of the repressive influence of Gt, and a consequent lack of repression (as per the model's predictions) in the region where Gt is expressed.

An important area of future improvements to our approach will be the quality and amount of data. The spatial expression profiles used here were obtained from manual parsing of stained (*in situ* hybridization) images, and are essentially qualitative. This is one of the reasons why our evaluations were based on correlation between expression patterns rather than more absolute measures of prediction accuracy. More accurate quantifications that are under way [61] should lead to improved analysis. Our approach assumes that the expression profiles of TFs and CRMs were synchronized (from the same developmental time), although this is not entirely true: the temporal resolution of the data set is not high enough to ensure such synchronization, and this is another direction where future, higher resolution data sets will be needed. Moreover, since we do not have data characterizing the dynamic state of chromatin (nucleosome distributions and their chemical modifications), we did not explicitly model the changes of chromatin structure that may be induced by TF association. With more high-quality expression data and ideally more epigenetic data as well, it should be possible to extend our models with additional details and to incorporate theoretical models of chromatin structure [62], [63].

### Broader applications

The models presented here are intended to be usable in a variety of regulatory systems in different species. It is true however that a regulatory system would need to be very well understood at a qualitative level and characterized by quantitative measurements at multiple levels, before we can apply such models. We would need the following information to train the models: (1) the expression readouts of a set of promoters or CRMs, (2) a reasonably complete set of TFs involved in the regulatory network, (3) quantification of their concentration profiles, and (4) their binding specificities. At this time, such a data set is often not available, making it difficult to evaluate the generalizability of the models.

A promising application of the proposed quantitative models lies in the prediction and characterization of novel CRMs. Once a sequence-expression model is trained, it may be applied genome-wide to predict segments that have the potential to direct the expression patterns of neighboring genes. The model may also be used to predict the effect of individual transcription factor perturbations, leading us to individual TF-CRM interactions. This paradigm requires quantitative measurements of TF levels, a requirement that may be mitigated to some extent by using mRNA levels of TF genes, but ideally by direct protein level measurements. Recent developments in proteomics and in high-throughput assays of post-translational modifications offer great hope in providing the necessary TF activity data [64].

The models offer new ways to approach the study of regulatory sequence evolution. Transcription factor binding sites have been reported to undergo frequent loss and gain, but it is not clear what the functional consequences of these changes are. We saw an example of how the functional context provided by the model may be combined with cross-species sequence comparison to provide new insights into binding site turnover. In general, sequence-expression models allow us to predict the changes in expression pattern that result from any evolutionary change at the sequence level. This interpretative power may be harnessed to investigate how regulatory sequences evolve under different schemes of selection, and begin to answer questions such as “With gene expression under purifying selection, how tolerant is a sequence to the gain and loss of binding sites?” or “How feasible is it to evolve a novel expression pattern using only simple nucleotide level changes, i.e., substitutions, insertions and deletions?” [65].

Quantitative models have a natural relevance in the field of synthetic biology. In order to design gene networks with a well-defined input/output characterization, we need the ability to engineer gene promoters or enhancers that direct specific expression patterns (outputs) in response to the specific levels of the regulators (inputs). This ability in turn requires a tool to predict the expression pattern corresponding to any given sequence. Moreover, to search in a very large sequence space, an efficient sequence-to-expression mapping will be crucial. This will be a place where our dynamic programming-based algorithms make a large difference.

In the long run, we expect quantitative models to be able to consider for example the entire intergenic region next to a gene (and not only individual CRMs) and predict the gene's spatial-temporal expression pattern. The GEMSTAT models are an important preliminary step towards this grand goal.

## Supporting Information

Figure S1 Comparison of two models of synergistic activation. (A) Cooperative Binding model: cooperative interactions between adjacent bound TF molecules, the transcriptional effects (interaction with BTM) of multiple TF molecules are additive. (B) Multiplicative Activation model: the transcriptional effects of multiple TF molecules are multiplicative, no cooperative interactions between adjacent bound TF molecules. The x-axis is the weight of a single site, q (thus q = 1 corresponds to occupancy of a single site 1/2), which is proportional to the concentration of the transcriptional activator, A. Note the two models predict the same expression for any given [A] at n = 1, but the relative level at larger n is different under the two models.
(0.09 MB PDF)

Figure S2 Predicted expression profiles of a DirectInt model with no cooperativity (“no-coop”, blue) and a model with self-cooperative binding for Bcd and Kni (“coop”, green) are shown for each CRM, with reference to the CRM's known readout (“Obs.”, red). The correlation coefficient between a model's prediction and the known readout is indicated in the top right corner of the panel. Each expression profile is on a scale of 0 to 1 (scaling does not affect correlation coefficient), and shown for bins 20 to 80 (i.e., 80% e.l. to 20% egg length) of the embryo. The CRM's name is color coded to indicate the better model (green for “coop”, and blue for “no-coop”), i.e., CC>0.65, difference in CC>0.05. All 37 CRMs in the data set are shown here.
(0.10 MB PDF)

Figure S3 Comparison with Segal et al [5]. The predictions of the DirectInt-Coop model (with homotypic cooperative interactions of Bcd and Kni), using CRMs, factor concentration profiles, and motifs from Segal et al., are shown in blue, along with observed expression patterns (red); as well as predicted expression patterns from Segal et al. (green). The average CC over all 44 CRMs was 0.591 under the DirectInt-Coop model and 0.579 under the Segal model. However, this is not a rigorous comparison of the two models, for multiple reasons: (1) the motifs used by both models were obtained by Segal et al. so as to optimize the performance of their model; we used those motifs without further tuning, and (2) our optimization used average CC (the measure of evaluation) as the objective function, while the Segal model was optimized for sum of squared errors.
(0.17 MB PDF)

Figure S4 Effect of transcriptional synergy (multiplicative effect of multiple activator molecules) on model performance in the absence of cooperative DNA binding of TFs. Semantics of the plots are as in Figure 2, with the only difference being that the models being compared here are one with transcriptional synergy (“synergy”, green) and one without (“nosynergy”, blue). Shown are all CRMs where the multiplicative model is better than or worse than the additive model (CC>0.65, difference in CC>0.05). As in Figure 2, CRM labels are color coded to indicate the better model. Evaluations are for a DirectInt model in the absence of self-cooperative DNA binding.
(0.06 MB PDF)

Figure S5 Effect of transcriptional synergy (multiplicative effect of multiple activators) on model performance in the presence of cooperative DNA binding of TFs. This is similar to Figure S4, except that evaluations are for a DirectInt model with Bcd and Cad self-cooperative DNA-binding.
(0.05 MB PDF)

Figure S6 Evaluation of short-range repression model. These are the same results for Gt and Kni, as in Figure 4AB.
(0.08 MB PDF)

Figure S7 Predicted expression profile of the Kr-SRR model (green) is compared to that of the DirectInt model (DI, blue), with reference to the known expression readout (red). Also shown is the predicted profile of the Kr-SRR-Kr- model (green dashed line), where Kr has been knocked down to reveal the contribution that Kr-driven repression makes to the profile of the Kr-SRR model. Shown are all of the CRMs where the Kr-SRR model had CC>0.65, a CC improvement of more than 0.05 over the corresponding “knock down” model (Kr-SRR-Kr-) and was either better than or roughly as accurate (difference in CC<0.05) as the DirectInt model.
(0.05 MB PDF)

Figure S8 Predicted expression profile of the Hb-SRR model (green) is compared to that of the DirectInt model (DI, blue), with reference to the known expression readout (red). Also shown is the predicted profile of the Hb-SRR-Hb- model (green dashed line), where Hb has been knocked down to reveal the contribution that Hb-driven repression makes to the profile of the Hb-SRR model. Shown are all of the CRMs where the Hb-SRR model had CC>0.65, a CC improvement of more than 0.05 over the corresponding “knock down” model (Hb-SRR-Hb-) and was either better than or roughly as accurate (difference in CC<0.05) as the DirectInt model.
(0.03 MB PDF)

Table S1 Model parameters.
(0.03 MB DOC)

Table S2 Comparison of models with or without cooperative DNA binding by TFs.
(0.04 MB DOC)

Table S3 Comparison of models with or without synergistic transcriptional activation.
(0.03 MB DOC)

Text S1 Additional results and details of the methods.
(0.19 MB PDF)
