# Supplementary figures and images for: Thermodynamics-Based Models of Transcriptional Regulation by Enhancers: The Roles of Synergistic Activation, Cooperative Binding and Short-Range Repression
Source: PLoS Comput Biol. 2010 Sep 16;6(9):e1000935. doi: 10.1371/journal.pcbi.1000935 (PMC2940721; doi:10.1371/journal.pcbi.1000935)

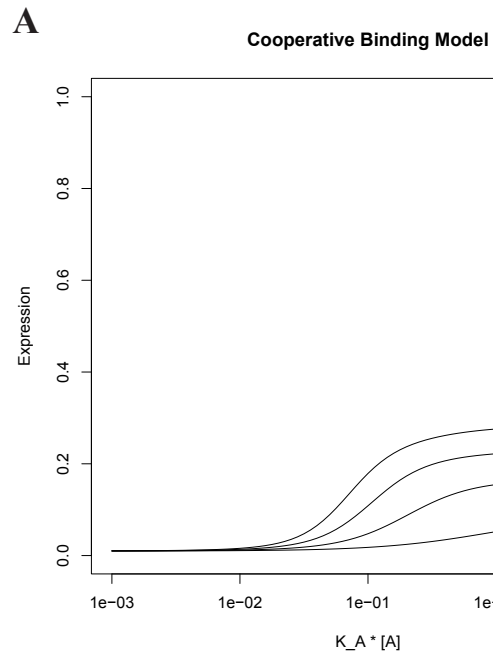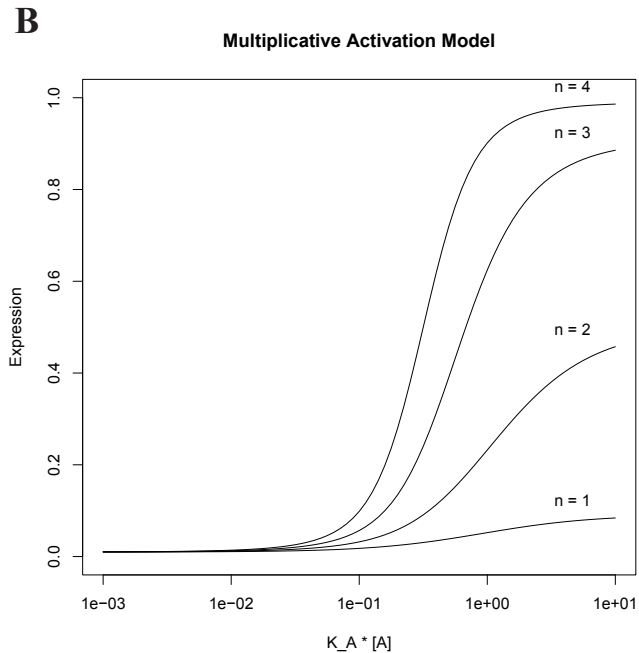

Supplement: Figure S1 — Comparison of two models of synergistic activation. (A) Cooperative Binding model: cooperative interactions between adjacent bound TF molecules, the transcriptional effects (interaction with BTM) of multiple TF molecules are additive. (B) Multiplicative Activation model: the transcriptional effects of multiple TF molecules are multiplicative, no cooperative interactions between adjacent bound TF molecules. The x-axis is the weight of a single site, q (thus q = 1 corresponds to occupancy of a single site 1/2), which is proportional to the concentration of the transcriptional activator, A. Note the two models predict the same expression for any given [A] at n = 1, but the relative level at larger n is different under the two models. (0.09 MB PDF) [file pcbi.1000935.s001.pdf]

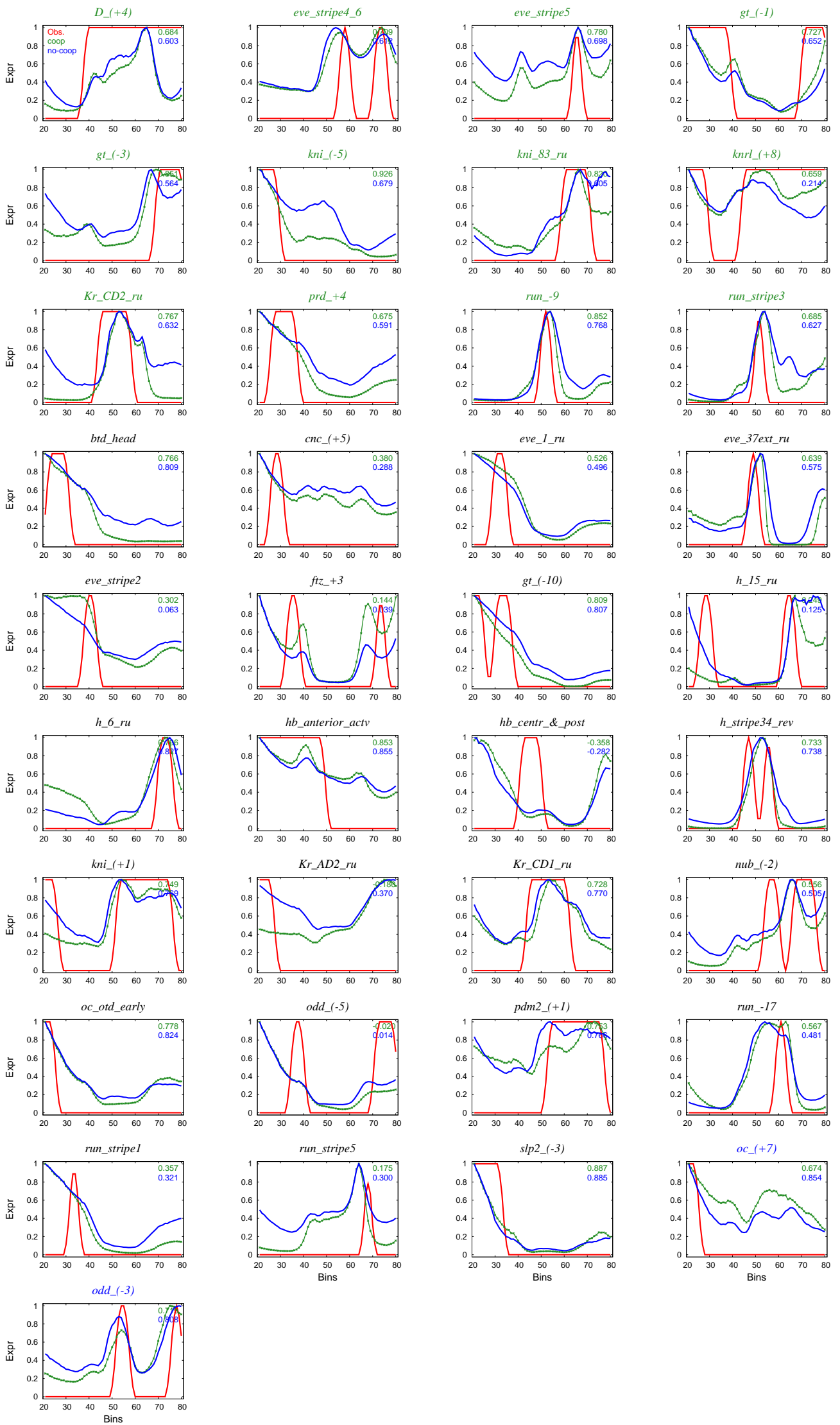

Supplement: Figure S2 — Predicted expression profiles of a DirectInt model with no cooperativity (“no-coop”, blue) and a model with self-cooperative binding for Bcd and Kni (“coop”, green) are shown for each CRM, with reference to the CRM's known readout (“Obs.”, red). The correlation coefficient between a model's prediction and the known readout is indicated in the top right corner of the panel. Each expression profile is on a scale of 0 to 1 (scaling does not affect correlation coefficient), and shown for bins 20 to 80 (i.e., 80% e.l. to 20% egg length) of the embryo. The CRM's name is color coded to indicate the better model (green for “coop”, and blue for “no-coop”), i.e., CC>0.65, difference in CC>0.05. All 37 CRMs in the data set are shown here. (0.10 MB PDF) [file pcbi.1000935.s002.pdf]

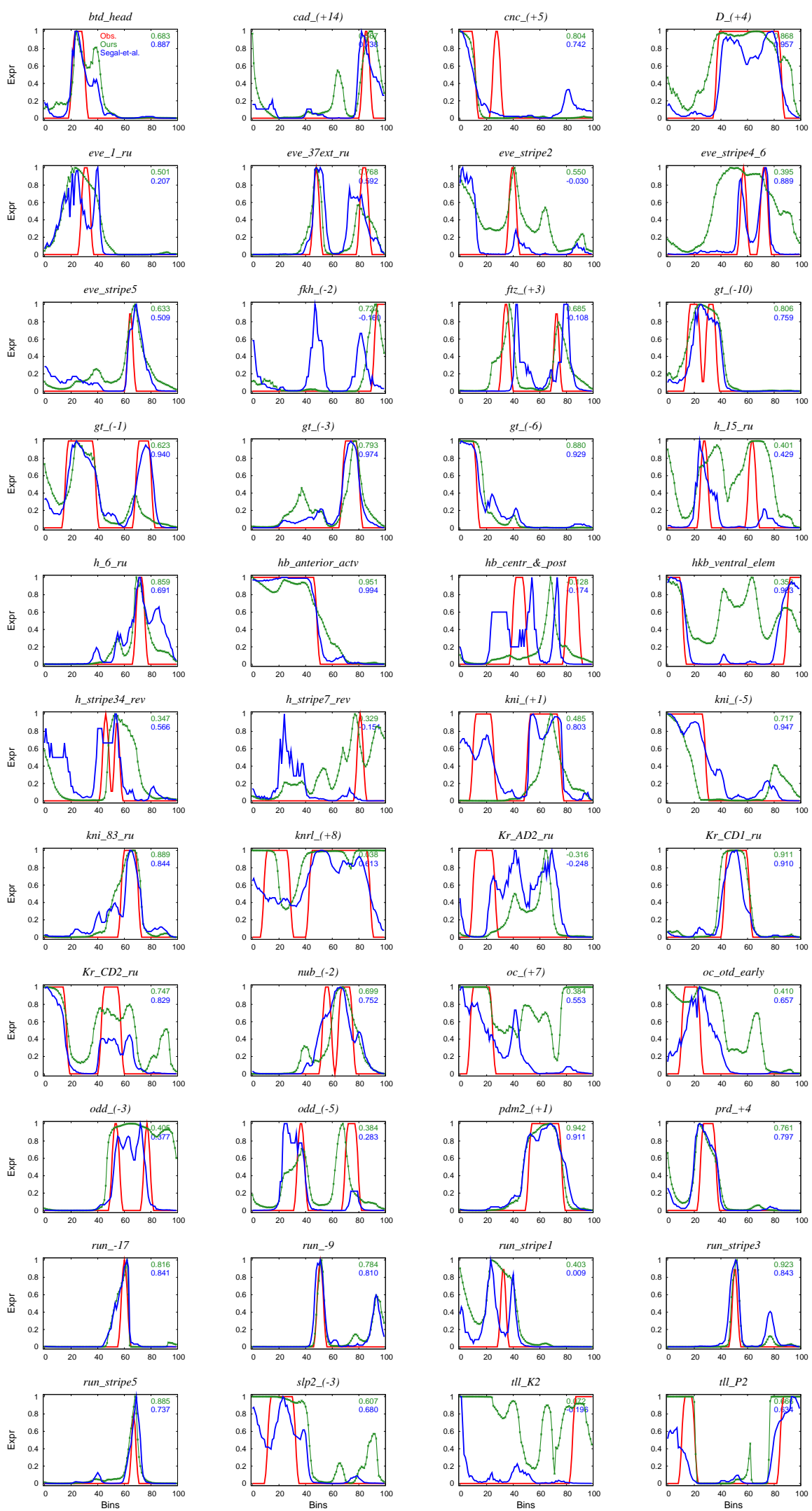

Supplement: Figure S3 — Comparison with Segal et al [5]. The predictions of the DirectInt-Coop model (with homotypic cooperative interactions of Bcd and Kni), using CRMs, factor concentration profiles, and motifs from Segal et al., are shown in blue, along with observed expression patterns (red); as well as predicted expression patterns from Segal et al. (green). The average CC over all 44 CRMs was 0.591 under the DirectInt-Coop model and 0.579 under the Segal model. However, this is not a rigorous comparison of the two models, for multiple reasons: (1) the motifs used by both models were obtained by Segal et al. so as to optimize the performance of their model; we used those motifs without further tuning, and (2) our optimization used average CC (the measure of evaluation) as the objective function, while the Segal model was optimized for sum of squared errors. (0.17 MB PDF) [file pcbi.1000935.s003.pdf]

*eve\_stripe5*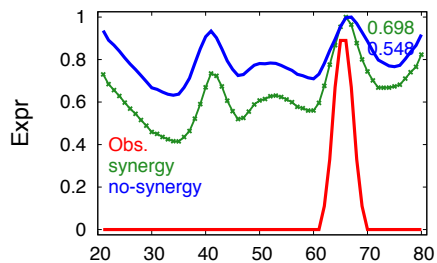*kni\_(-5)*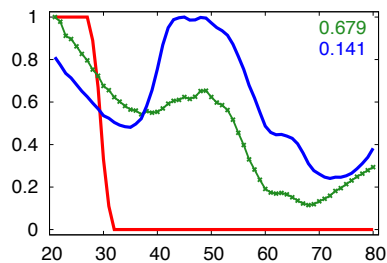*oc\_(+7)*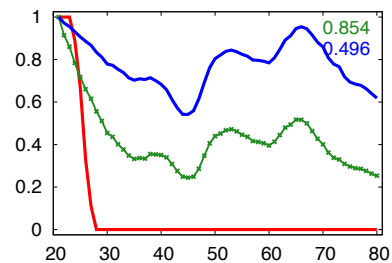*oc\_otd\_early*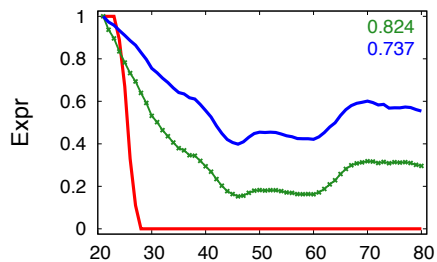*odd\_(-3)*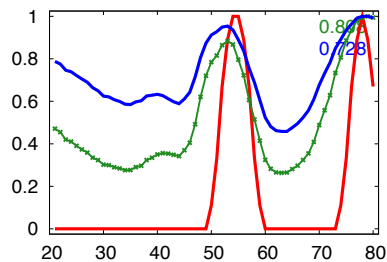*slp2\_(-3)*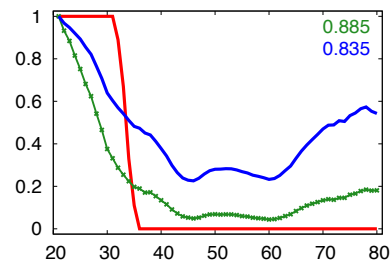*D\_(+4)*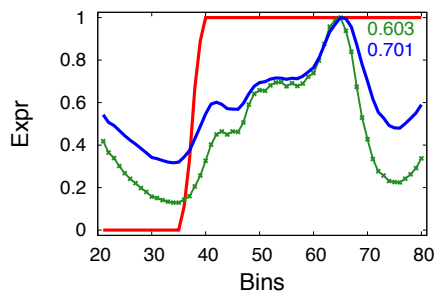*gt\_(-3)*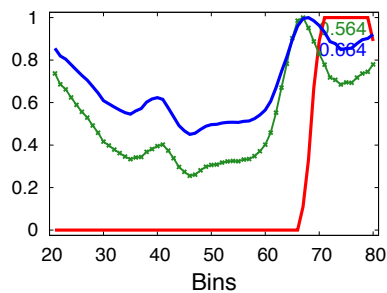*Kr\_CD2\_ru*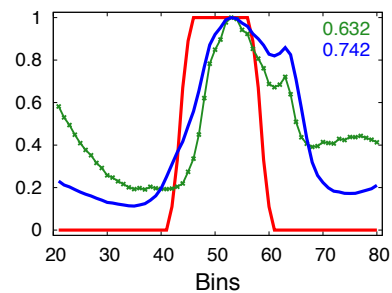

Supplement: Figure S4 — Effect of transcriptional synergy (multiplicative effect of multiple activator molecules) on model performance in the absence of cooperative DNA binding of TFs. Semantics of the plots are as in Figure 2, with the only difference being that the models being compared here are one with transcriptional synergy (“synergy”, green) and one without (“nosynergy”, blue). Shown are all CRMs where the multiplicative model is better than or worse than the additive model (CC>0.65, difference in CC>0.05). As in Figure 2, CRM labels are color coded to indicate the better model. Evaluations are for a DirectInt model in the absence of self-cooperative DNA binding. (0.06 MB PDF) [file pcbi.1000935.s004.pdf]

*btd\_head*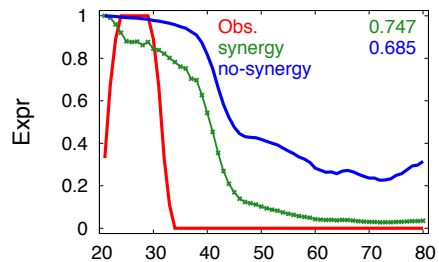*eve\_stripe4\_6*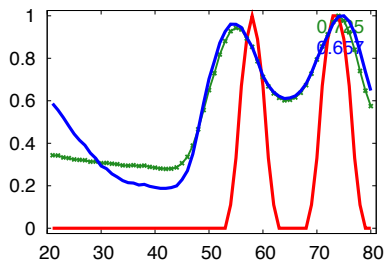*hb\_anterior\_actv*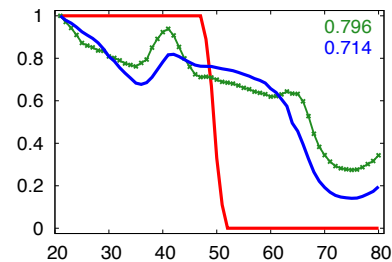*kni\_83\_ru*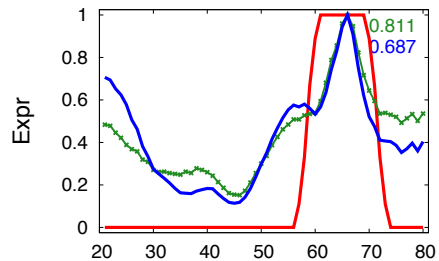*oc\_(+7)*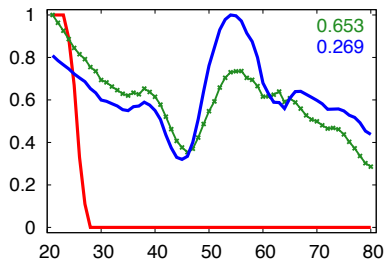*oc\_otd\_early*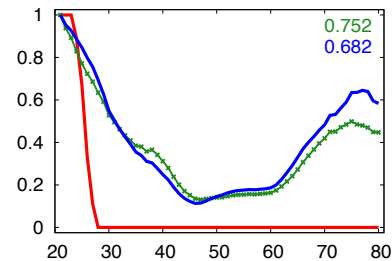*pdm2\_(+1)*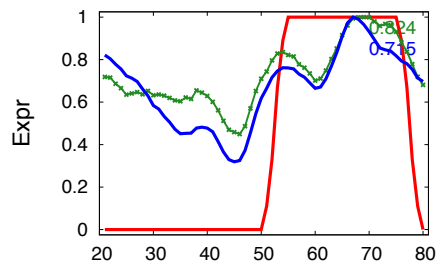*slp2\_(-3)*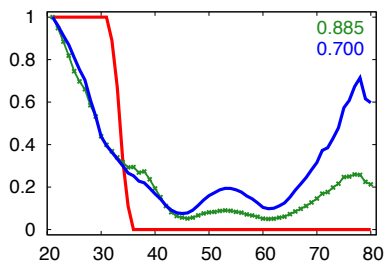

Bins

Bins

Supplement: Figure S5 — Effect of transcriptional synergy (multiplicative effect of multiple activators) on model performance in the presence of cooperative DNA binding of TFs. This is similar to Figure S4, except that evaluations are for a DirectInt model with Bcd and Cad self-cooperative DNA-binding. (0.05 MB PDF) [file pcbi.1000935.s005.pdf]

**A**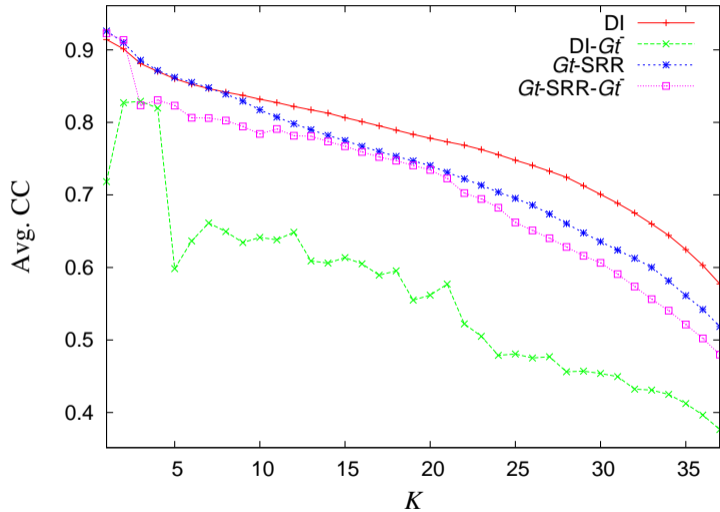**B**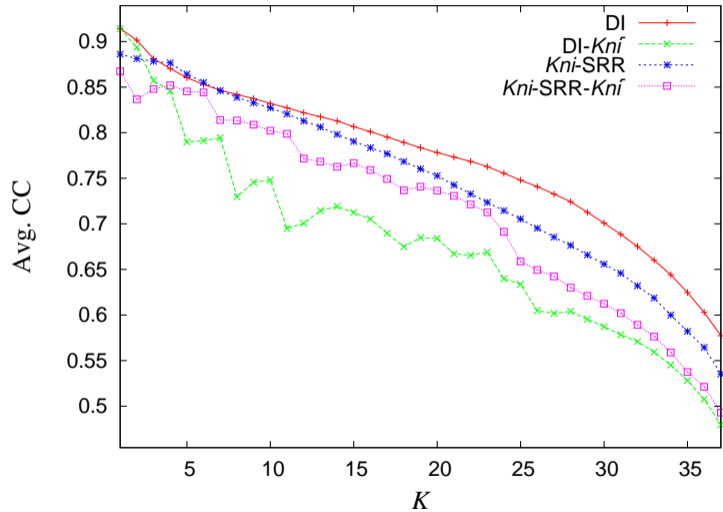

Supplement: Figure S6 — Evaluation of short-range repression model. These are the same results for Gt and Kni, as in Figure 4AB. (0.08 MB PDF) [file pcbi.1000935.s006.pdf]

*gt\_(-1)*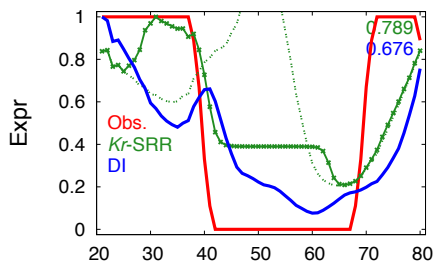*h\_6\_ru*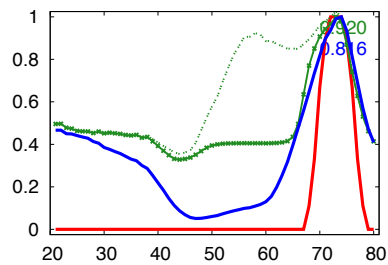*kni\_83\_ru*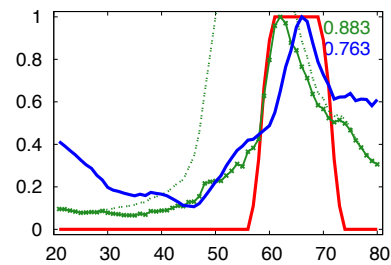*gt\_(-10)*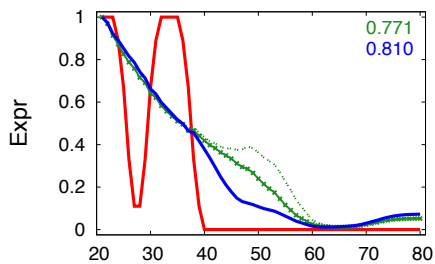*kni\_(+1)*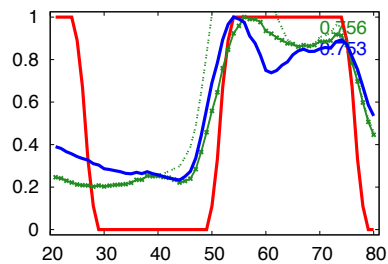*prd\_+4*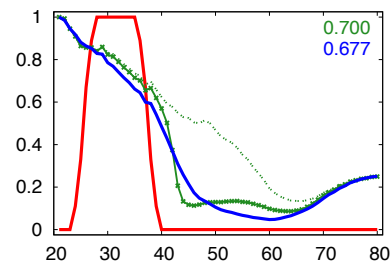*slp2\_(-3)*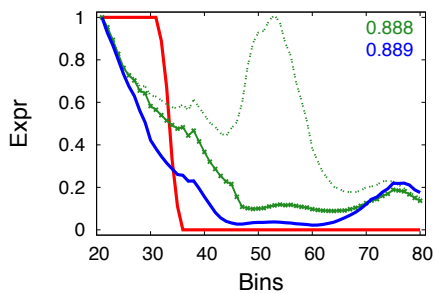

Supplement: Figure S7 — Predicted expression profile of the Kr-SRR model (green) is compared to that of the DirectInt model (DI, blue), with reference to the known expression readout (red). Also shown is the predicted profile of the Kr-SRR-Kr- model (green dashed line), where Kr has been knocked down to reveal the contribution that Kr-driven repression makes to the profile of the Kr-SRR model. Shown are all of the CRMs where the Kr-SRR model had CC>0.65, a CC improvement of more than 0.05 over the corresponding “knock down” model (Kr-SRR-Kr-) and was either better than or roughly as accurate (difference in CC<0.05) as the DirectInt model. (0.05 MB PDF) [file pcbi.1000935.s007.pdf]

*eve\_stripe4\_6*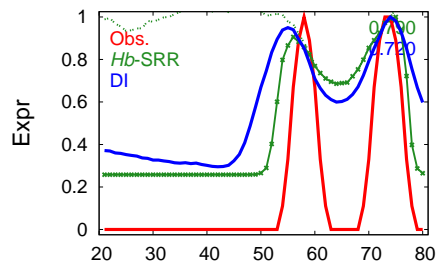*knrl\_(+8)*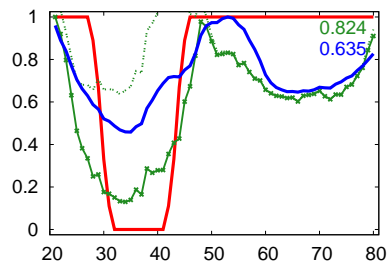*Kr\_CD1\_ru*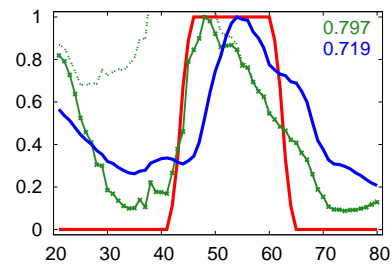*oc\_(+7)*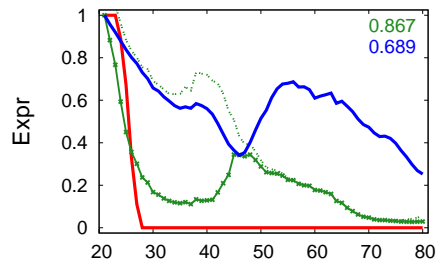*oc\_otd\_early*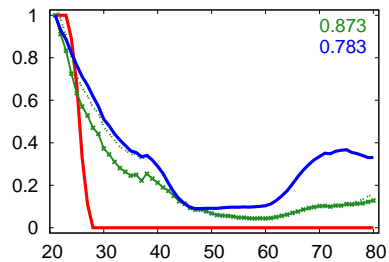*gt(-1)*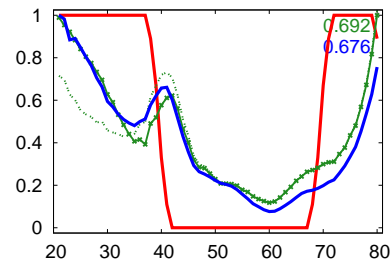*h\_6\_ru*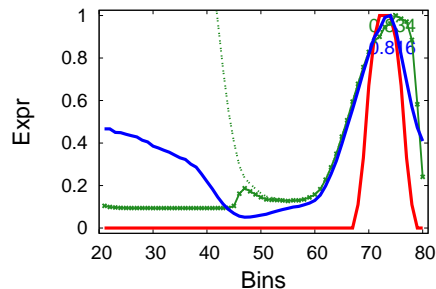*kni\_(+1)*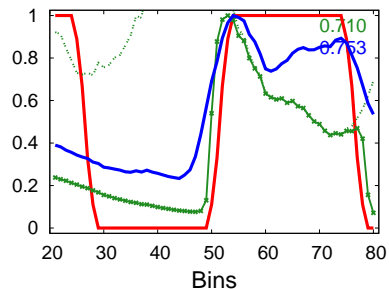*kni\_83\_ru*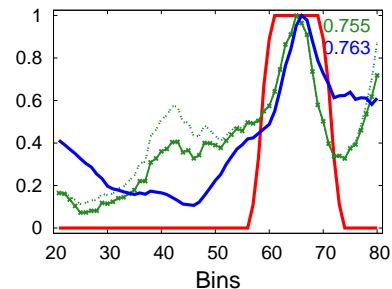

Supplement: Figure S8 — Predicted expression profile of the Hb-SRR model (green) is compared to that of the DirectInt model (DI, blue), with reference to the known expression readout (red). Also shown is the predicted profile of the Hb-SRR-Hb- model (green dashed line), where Hb has been knocked down to reveal the contribution that Hb-driven repression makes to the profile of the Hb-SRR model. Shown are all of the CRMs where the Hb-SRR model had CC>0.65, a CC improvement of more than 0.05 over the corresponding “knock down” model (Hb-SRR-Hb-) and was either better than or roughly as accurate (difference in CC<0.05) as the DirectInt model. (0.03 MB PDF) [file pcbi.1000935.s008.pdf]
