# Supplementary material for: Thermodynamics-Based Models of Transcriptional Regulation by Enhancers: The Roles of Synergistic Activation, Cooperative Binding and Short-Range Repression
Source: PLoS Comput Biol. 2010 Sep 16;6(9):e1000935. doi: 10.1371/journal.pcbi.1000935 (PMC2940721; doi:10.1371/journal.pcbi.1000935)
Supplement: Table S1 — Model parameters. (0.03 MB DOC) [file pcbi.1000935.s009.doc]

**Table S1.** A list of parameters used by various models in GEMSTAT. Certain parameters are specific to the DirectInt or the SRR model, while others are common to both models.

| **Model choice** | **Parameter** | **How many?** | **Description** | **Training**  **Allowed?** |
| --- | --- | --- | --- | --- |
| DirectInt | α | One per TF | Strength of TF’s effect on promoter occupancy. | Yes |
| SRR | α | One per activator | Strength of TF’s activation effect. | Yes |
| SRR | β | One per repressor | Strength of TF’s repression (quenching) effect. | Yes |
| SRR | d_R_ | One overall | Range (in bp) of repressor effect. | No |
| DirectInt/SRR | N_MA_ | One overall | Number of bound activators that may simultaneously interact with BTM. | No |
| DirectInt/SRR | DNA binding | One per TF | Equal to *K(S_max_*)ν in Equation 2. | Yes |
| DirectInt/SRR | ω | One per TF that exhibits self-cooperative DNA binding | Strength of protein-protein interaction between two bound molecules of the TF (cooperative binding). | Yes |
| DirectInt/SRR | d_C_ | One overall | Range within which cooperative binding is allowed. | No |
| DirectInt/SRR | q_BTM_ (Promoter efficiency) | One overall | Basal expression level. | Yes |
