# Supplementary material for: Thermodynamics-Based Models of Transcriptional Regulation by Enhancers: The Roles of Synergistic Activation, Cooperative Binding and Short-Range Repression
Source: PLoS Comput Biol. 2010 Sep 16;6(9):e1000935. doi: 10.1371/journal.pcbi.1000935 (PMC2940721; doi:10.1371/journal.pcbi.1000935)
Supplement: Table S2 — Comparison of models with or without cooperative DNA binding by TFs. (0.04 MB DOC) [file pcbi.1000935.s010.doc]

**Table S2.** We calculated the average correlation coefficients under seven independent replicates of 10-fold cross-validation on the 37 CRMs. Each replicate represents a different, random parition of the 37 CRMs into 10 “folds”. The “cross validation correlation coefficient” (CVCC) for six of these replicates (“Data set 1”, “Data set 2”, etc.) is shown here, while the same for the seventh replicate is shown in Table 3. The row “No Coop” shows the calculated values of the DirectInt model with no cooperative interactions. Each row named by a single TF represents the results of the DirectInt-Coop model with homotypic cooperative interaction of that TF (but no other TFs). The penultimate row shows the performance of the model that allows homotypic cooperativity for the entire set of six TFs. The last row reports the results from using both *Bcd* and *Kni* as homotypic cooperative factors.

| **Model** | **Set 1** | **Set 2** | **Set 3** | **Set 4** | **Set 5** | **Set 6** |
| --- | --- | --- | --- | --- | --- | --- |
| No Coop | 0.387 | 0.379 | 0.382 | 0.346 | 0.398 | 0.428 |
| *Bcd* Coop | 0.427 | 0.447 | 0.409 | 0.442 | 0.433 | 0.444 |
| *Cad* Coop | 0.451 | 0.416 | 0.462 | 0.423 | 0.450 | 0.449 |
| *Gt* Coop | 0.399 | 0.385 | 0.427 | 0.437 | 0.369 | 0.417 |
| *Hb* Coop | 0.402 | 0.421 | 0.451 | 0.381 | 0.410 | 0.398 |
| *Kni* Coop | 0.432 | 0.418 | 0.434 | 0.458 | 0.416 | 0.442 |
| *Kr* Coop | 0.444 | 0.424 | 0.471 | 0.456 | 0.462 | 0.437 |
| All TF Coop | 0.359 | 0.428 | 0.353 | 0.402 | 0.405 | 0.401 |
| *Bcd-Kni* Coop | 0.413 | 0.431 | 0.438 | 0.420 | 0.387 | 0.416 |
