# Supplementary material for: Thermodynamics-Based Models of Transcriptional Regulation by Enhancers: The Roles of Synergistic Activation, Cooperative Binding and Short-Range Repression
Source: PLoS Comput Biol. 2010 Sep 16;6(9):e1000935. doi: 10.1371/journal.pcbi.1000935 (PMC2940721; doi:10.1371/journal.pcbi.1000935)
Supplement: Table S3 — Comparison of models with or without synergistic transcriptional activation. (0.03 MB DOC) [file pcbi.1000935.s011.doc]

**Table S3.** We compared the alternative approaches to model combinations of transcriptional synergy and cooperativity by calculating the CVCC of the cross validation replicates of Table S1. In modeling synergy, ‘N’ represents setting N_MA_ = 1 and ‘Y’ corresponds to N_MA_ = ∞. ‘Y’ for cooperativity denotes a model with the inclusion of homotypic cooperative interactions for *Bcd* and *Cad*, whereas ‘N” represents a model with no cooperative interactions.

| **Synergy** | **Coop** | **Set 1** | **Set 2** | **Set 3** | **Set 4** | **Set 5** | **Set 6** |
| --- | --- | --- | --- | --- | --- | --- | --- |
| N | N | 0.252 | 0.281 | 0.281 | 0.268 | 0.322 | 0.295 |
| Y | N | 0.387 | 0.379 | 0.382 | 0.346 | 0.398 | 0.428 |
| N | Y | 0.274 | 0.251 | 0.258 | 0.260 | 0.246 | 0.292 |
| Y | Y | 0.391 | 0.406 | 0.430 | 0.452 | 0.376 | 0.396 |
