# Supplementary material for: Thermodynamics-Based Models of Transcriptional Regulation by Enhancers: The Roles of Synergistic Activation, Cooperative Binding and Short-Range Repression
Source: PLoS Comput Biol. 2010 Sep 16;6(9):e1000935. doi: 10.1371/journal.pcbi.1000935 (PMC2940721; doi:10.1371/journal.pcbi.1000935)
Supplement: Text S1 — Additional results and details of the methods. (0.19 MB PDF) [file pcbi.1000935.s012.pdf]

Text S1: Supplementary Methods and Results

Comparison of two mechanisms of synergistic activation

Many promoter and enhancer sequences harboring more than one activator site exhibit synergistic activation: the effect of multiple sites is more than the sum of the effects of individual sites [1,2]. In the text, we discussed two possible mechanisms of such synergistic activation: (1) cooperative binding of TF molecules to DNA sequences; and (2) multiplicative activation (see the section "Modeling the action of multiple activators") possibly due to the simultaneous interaction of multiple TF molecules with the basal transcriptional machinery (BTM). In this section, we discuss how these two mechanisms may lead to qualitatively different behavior. To compare the two models, we assume that in each model the effect of the other model is absent, i.e., no multiplicative activation in (1), and no cooperative interaction between TF molecules in (2).

We consider a simple case of one sequence with n identical binding sites of transcription factor A. The behavior of the sequence is characterized by how the transcriptional response varies with the activator concentration, denoted as [A]. We define the Boltzmann weight of a single site as $q = K_A[A]$ where K_A is the association constant of the site to A. The occupancy of a single site (the probability of binding), without interaction with any other sites, is thus $1/(1+q)$. The physiological range of q could be very broad, from much less (very weak site or very low TF concentration) to much greater than 1 (saturation of occupancy). We denote by α the transcriptional effect of a single bound molecule of A, and by ω the cooperative interaction between two bound molecules of A, assuming that the strength of cooperative interaction is independent of spatial arrangement of sites (for more description of these parameters, see the description of DirectInt model in the Methods section). Furthermore we have one more parameter, q_{BTM}, for the basal level of interaction between the BTM and the core promoter sequence. Below we give the explicit equations of the expression level as functions of other parameters, for two activation models.

Cooperative Binding (CB) model

We first consider the case where there is cooperative interaction between two TF molecules bound to adjacent sites in the DNA sequence, but no multiplicative activation (i.e., the transcriptional effects of multiple bound activator molecules are additive). Under this model, any two molecules of A bound at adjacent sites can interact with each other; thus in a configuration where i sites are occupied, there will be i TF-DNA interactions (one per site), and i-1 cooperative interactions (one per consecutive pair of sites). We have:

$$Z_{OFF} = 1 + \sum_{i=1}^{n} \binom{n}{i} q^i \omega^{i-1} = 1 + \frac{(1+\omega q)^n - 1}{\omega} \qquad \text{(S1)}$$

Under the additive activation model, the configuration where i sites are occupied has $Q(i) = i\alpha$, i.e., at any time, only one activator molecule is allowed to interact with BTM:

$$Z_{ON} = q_{BTM}[1 + \sum_{i=1}^{n} \binom{n}{i} q^i \omega^{i-1} i\alpha] = q_{BTM}(1 + n\alpha q(1+\omega q)^{n-1}) \qquad \text{(S2)}$$

The expression level is given by Equation (1) in the main text.

Multiplicative Activation (MA) model

We next consider the case where interactions of TF molecules and BTM are multiplicative, but there is no cooperative interaction between TF molecules. When the BTM is not present, we simply have:

$$Z_{OFF} = (1+q)^n \tag{S3}$$

Under the multiplicative activation model, the configuration where i sites are occupied has $Q(i) = \alpha^i$. We have:

$$Z_{ON} = q_{BTM}[1+\sum_{i=1}^{n}\binom{n}{i}q^i\alpha^i] = q_{BTM}(1+q\alpha)^n \tag{S4}$$

Comparison of the two synergistic activation models

For simplicity of discussion, we define: $\eta = Z_{ON} / Z_{OFF}$, and then the expression level is proportional to the occupancy of promoter, which is: $E = \eta / (1+\eta)$. When $\eta \ll 1$ (which may often be the case due to the small value of the q_{BTM} term), $E \approx \eta$, thus we can approximate expression output of the sequence using η. So we will analyze how η varies with the number of sites n under two models. We start with the MA model, where the value of η is given by the simple equation:

$$\eta_n = q_{BTM}\left(\frac{1+q\alpha}{1+q}\right)^n \tag{S5}$$

Thus under the MA model, the expression output is roughly exponential in the number of sites. Since α is always large than 1 for activators, the base term is larger than 1. When it reaches a significant value (e.g., due to a high q resulting from high concentration [A] or a high α), we could easily have the synergistic effect: $\eta_2 \gg 2\eta_1$.

For the CB model, we claim that there will be no synergistic effect at high/saturating concentration of A. To see this, we note that when $\omega q \gg 1$ due to high [A] (or high cooperativity, or strong site affinity), the equations of Z_{OFF} and Z_{ON} can be simplified, we have:

$$\eta_n \approx \frac{q_{BTM} n\alpha q\omega^{n-1}q^{n-1}}{\omega^{n-1}q^n} = q_{BTM} n\alpha \tag{S6}$$

Therefore, the expression output is only roughly linear to the number of sites at high [A]. This conclusion should be intuitively clear: cooperative binding mainly facilitates occupancy of TF molecules to the DNA sequence, but at high [A], the occupancy of any single binding site is already close to 1, thus cooperativity from additional sites will not produce further benefit.

In summary, the MA and CB models have qualitatively different dosage-response behavior: the MA model leads to the strongest transcriptional synergy at high concentrations of TF, and low synergy at low TF concentration; on the contrary, the CA model leads to almost complete absence of synergy at high TF concentration. This difference of behavior is illustrated in Figure S1, where the expression output is plot against [A] (scaled by $1/K_A$). The results were obtained at $\alpha = 10.0$, $\omega = 20.0$ and $q_{BTM} = 0.01$. Different values of these parameters (within biologically realistic range) give similar qualitative results. At $q = [A]K_A = 1$, the occupancy of a single site is $1/(1+q) = 1/2$, and the occupancy is nearly full at $q = 10$. It is clear that the synergistic behaviors of the two models are very different at large values of q. Also note that at high [A],

while n = 2 or 3 leads to high synergism comparing with n = 1, as n further increases, the synergistic effect is diminished as the sequence approaches saturation (of BTM occupancy).

Details of thermodynamic models and algorithms

The statistical weight of individual binding sites

For a single site S, the statistical weight due to TF binding is given by $q(S) = [TF]K(S_{\max})e^{-\beta\Delta E(S)}$, where [TF] is the concentration of the TF, S_{max} is the strongest binding site (the consensus sequence) of this TF, $K(S_{max})$ is the effective association constant of S_{max}, ΔE(S) is the "mismatch energy" of the site S relative to S_{max} and β is the Boltzmann constant. According to the theory of Berg & von Hippel [3,4], the mismatch energy is related to the log likelihood ratio (LLR) score of a site by $\beta\Delta E(S) = -LLR(S) + LLR(S_{\max})$, where $LLR(\cdot)$ is computed from the known position weight matrix (PWM) of the TF and the background distribution of nucleotides [4]. Since our input data includes relative values for TF concentration (on an arbitrary scale), we rewrite [TF] above as $\nu[TF]_{rel}$ where $[TF]_{rel}$ is concentration relative to some unknown reference, and ν is the value of this reference level. The statistical weight of a site can thus be computed as

$$q(S) = K(S_{\max})\nu[TF]_{rel}\exp[LLR(S) - LLR(S_{\max})] \tag{S7}$$

where ($\nu K(S_{max})$) is a free parameter, $[TF]_{rel}$ is the given (relative) TF concentration value, and $LLR(\cdot)$ is computable from the PWM.

Cooperative interaction of transcription factor molecules

In general, the statistical weight $\omega_{AB}(d)$ due to the interaction between two bound TF molecules A and B depends on their distance d. It is not known *a priori* what a suitable functional description of $\omega_{AB}(d)$ should be. For the results presented in this paper (when cooperative interactions are involved), we use a simple binary function for $\omega_{AB}(d)$, i.e. it is some constant ω_{AB} if d is less than or equal to some threshold d_C, and 1 otherwise (no interaction). The parameter ω_{AB} is treated as a free parameter of the model, dependent on A and B. Our default value of d_C is 50bp (many different values of d_C have been examined, and 50bp is the optimal value judged by the correlation coefficient (CC) of the DirectInt model with *Bcd* and *Kni* cooperativity). We also evaluated a Gaussian function with mean 0 (the interaction is maximum when the sites are close) and standard deviation s (a free parameter). This is effectively the same function used in Segal et al. [5], except that they examined only a single value of s (50bp) and our function will truncate (i.e. a constant value 1) if $d > d_C$. In spite of an extra parameter (s), we did not find evidence that the Gaussian form of cooperative interaction leads to higher CC than the simple binary form.

Dynamic Programming Formulation for SRR model with Unlimited Contact ($N_{MA} = \infty$)

In the short range repression model, $Z_{OFF}(i)$ can be split into two cases: $Z_0(i)$ where site i is in the bound-only state and $Z_1(i)$ where site i is bound-effective. Using these definitions, $Z_{OFF}(i) = Z_0(i) + Z_1(i)$. If i is bound-only, it could interact with other bound sites, but should not fall in the range of any effective repressor site. We have the following recurrence for $Z_0(i)$, where $d(i,j) > d_R$ enforces the constraint that no effective repressor site can be found within d_R of site i:

$$Z_0(i) = q(i)\left[\sum_{j\in\Phi(i)}\omega(i,j)Z_0(j) + \sum_{j<i,d(i,j)>d_R} Z_1(j) + 1\right] \tag{S8}$$

For $Z_1(i)$, we consider two cases: if $f(i)$ is an activator, then $Z_1(i) = 0$; otherwise, site i may interact with other bound-effective repressor sites, but no other bound-only sites should fall in the repression range of i. We have:

$$Z_1(i) = q(i)\beta_{f(i)} \left[\sum_{j \in \Phi(i)} \omega(i,j) Z_1(j) + \sum_{j<i, d(i,j)>d_R} Z_0(j) + 1 \right] \quad \text{(S9)}$$

where $\beta_{f(i)}$ is the repression strength of the repressor $f(i)$. To compute $Z_{ON}(i)$, we split the recursion into two cases, $Z_2(i)$ and $Z_3(i)$, corresponding to $Z_0(i)$ and $Z_1(i)$ respectively. Z_{ON} (i) = $Z_2(i)$ + $Z_3(i)$. $Z_2(i)$ incorporates the transcriptional effect, α, of the bound site i (1 if $f(i)$ is a repressor):

$$Z_2(i) = q(i)\alpha_{f(i)} \left[\sum_{j \in \Phi(i)} \omega(i,j) Z_2(j) + \sum_{j<i, d(i,j)>d_R} Z_3(j) + 1 \right] \quad \text{(S10)}$$

$Z_3(i)$ is defined only for repressor sites, and repressors do not interact with the BTM in this model.

$$Z_3(i) = q(i)\beta_{f(i)} \left[\sum_{j \in \Phi(i)} \omega(i,j) Z_3(j) + \sum_{j<i, d(i,j)>d_R} Z_2(j) + 1 \right] \quad \text{(S11)}$$

Again, $Z_{OFF} = \sum_i Z_{OFF}(i)$ and $Z_{ON} = \sum_i Z_{ON}(i)$. The time complexity of the algorithm is $O(n^2)$, where n is the number of sites. If we limit the range of cooperative interactions and repression to a constant value, e.g. 200 bp, the time complexity is $O(cn)$, where c is the average number of binding sites with this range.

Details of DirectInt model with limited contact for activation

To describe our activation model in the general case, we consider a configuration σ where N activator molecules (of the same type) are bound with the parameter N_{MA} indicating the maximum number of activator molecules that may simultaneously contact BTM. If $N \le N_{MA}$, then all bound activator molecules can simultaneously interact with BTM, we have $Q(\sigma) = (1+\alpha)^N$ where α is the transcriptional effect of the activator (note that each molecule may actually interact with BTM or not – imagine that the BTM-interaction site of an activator has two states, similar to TF binding sites in DNA, thus we have the term 1 here). If $N \ge N_{MA}$, then at most N_{MA} molecules can interact with BTM simultaneously. Since there are $\binom{N}{k}$ ways of choosing k molecules ($k \le N_{MA}$), this results in: $Q(\sigma) = \sum_{k=0}^{N_{MA}} \binom{N}{k} \alpha^k$, where α^k corresponds to the multiplicative effect of k molecules. If the bound activator molecules are of different types, the computation can be similarly achieved, by replacing binomial coefficients with multinomial coefficients, and α^k with another appropriate power term.

Our goal is to compute E (Equation (1)) from a given sequence incorporating limited contact of activation and still using dynamic programming. The computation of Z_{OFF} follows exactly the Equations (S2). At each configuration in this version of the DirectInt model, the number of activator molecules that interact simultaneously with BTM cannot exceed N_{MA}. To compute Z_{ON}, we define $Z_{ON}(i,k)$ as the sum of weights over all configurations where the site at i is bound and the number of contributing activators equals to k. Comparing with the algorithm where N_{MA} is unlimited, the additional index k is used to keep track of the number of BTM-interacting activator molecules. As before, $f(i)$ denotes the factor bound at site i. If $f(i)$ is an activator, it may or may not interact with BTM: contributing $\alpha_{f(i)}$ only if interaction occurs. Thus we have:

$$Z_{ON}(i,k) = q(i) \sum_{j \in \Phi(i)} \omega(i,j) Z_{ON}(j,k) + q(i)\alpha_{f(i)} \left[\sum_{j \in \Phi(i)} \omega(i,j) Z_{ON}(j,k-1) + [k=1] \right]$$
(S12)

The term *[k=1]* is the indicator function. When *f(i)* is a repressor, it will not contribute to activation:

$$Z_{ON}(i,k) = q(i)\alpha_{f(i)} \left[\sum_{j \in \Phi(i)} \omega(i,j) Z_{ON}(j,k) + [k=0] \right] \quad (S13)$$

When $k = 0$, the recurrences need to be modified: the terms containing $k\text{-}1$ will be removed. The final partition function is given by:

$$Z_{ON} = \sum_{i} \sum_{k=0}^{N_{MA}} Z_{ON}(i,k) \quad (S14)$$

The time complexity of the algorithm is $O(N_{MA}n^2)$, or $O(cN_{MA}n)$ if the range of cooperative interactions and repression is within a certain constant value, where c is the average number of binding sites with this range.

Implementation and model fitting

Overview

The program takes as input: a set of sequences, the PWMs of the relevant TFs and the expression patterns of the sequences and the TFs. It will estimate the model parameters that best explain the data, under any user specified model options. These options determine which of the several models to run, and the relevant model options. Specifically, the program supports two basic models: DirectInt and SRR models (see main text). The control options include: the role of a TF as an activator or repressor (only needed for the SRR model), the TF pairs with cooperative interactions, the distance thresholds for cooperative interactions (d_C) and short-range repression (d_R), and the multiplicative activation parameter N_{MA}.

The main steps of the programs are: extract the putative transcription factor binding sites in the input sequences, estimate parameters that optimize an objective function and print the results. Below, we describe the details of each step.

Binding site extraction

This step converts the DNA sequences into a representation of linear arrays of TFBSs, and also computes the LLR score of each TFBS. Since only TFBSs will be used in the computation, the future steps only operate on this representation. Transcription factor binding sites in each sequence were annotated as those having the log likelihood ratio (LLR) scores greater than 0.4 times the LLR score of the optimal site [6]. This threshold is weak enough to include a large number of putative sites for each TF, while keeping the running time low.

Objective function

For a given set of model parameters (see Table S1 for the list of parameters), our model computes the value of an objective function. We use the average correlation coefficient (Avg. CC) between

measured and predicted expressions of the input sequences. To avoid being trapped in the local maximums, we also use the sum of squared errors (SSE) during this optimization in an auxiliary fashion (see below). Suppose the input data set contain *n* sequences. Let *E(i)* and *P(i)* be the measured and the predicted expressions respectively for the *i*-th sequence S_i. Also, let *E(i,j)* and *P(i,j)* be the measured and the predicted expressions respectively of S_i under the *j*-th condition. Then for a given set of parameters Θ, the function Avg. CC is defined as

$$\sum_i r(\, E(i), P(i)|\Theta)/n,$$

where *r(X,Y)* denotes the correlation between two variables *X* and *Y*; and the function SSE is defined as

$$\sum_{i,j} [E(i,j) - P(i,j)|\Theta]^2.$$

Parameter optimization

We perform the optimization through multiple runs while alternating between Avg. CC and SSE as the objective function in these runs (starting with Avg. CC as the first objective function). We use a set of default parameters to start the first run. In subsequent runs, the program starts with the set of parameters that it learnt in the previous run. The optimization in a run of our program is achieved by alternating between the Nelder-Mead simplex method and the quasi-Newton method (the BFGS algorithm), both provided in the GNU Scientific Library [7]. Both the Nelder-Mead and the quasi-Newton algorithms are prone to producing sub-optimal solutions, and it is a common practice in such optimization problems to retrain the model with some randomly sampled values for the free parameters. Although the essence of random sampling is to find a new set of parameter values that can eventually lead us to a better solution, this approach is not guaranteed to improve the solution and we also did not find it to be helpful in our case. Our switching the objective function to SSE from Avg. CC in every other run was therefore an attempt to find a different set of starting parameters, which is essentially similar in spirit to doing a random sampling of the parameters. We found this scheme to be more helpful in searching the parameter space than random sampling.

Output

The program will output the best-fit values of the parameters shown in Table S1. These include the TF-specific parameters: for the DirectInt model, these are ($\nu K(S_{max})$) in Equation (2) and the transcriptional effect α; for the SRR model, these are ($\nu K(S_{max})$), α (for activators) and the repression effect β (for repressors). Since each TF is an activator or repressor in the SRR model, the number of free parameters per TF is two under either model. When cooperative interactions are specified, one free parameter is added for each cooperative pair. Here, we examine only homotypic cooperativity, hence we have up to one extra parameter per TF under the cooperative model. In addition, we have a parameter for the basal transcription by BTM, q_{BTM} .

Testing statistical significance of the difference of model predictions

We want to test if the difference between the predictions from two models is significant. To do this, we calculated the correlation coefficients of the predictions of the two models to the observed expression, $c_1(i) = r(E(i), P_1(i))$ and $c_2(i) = r(E(i), P_2(i))$, where *i* is the CRM index, E(i) is the observed expression profile, and $P_1(i)$, $P_2(i)$ are predicted expression profiles of the two models, respectively Using the uniform ratio distribution [8]

$$p_Z(z) = \begin{cases} \frac{1}{2} & 0 < z < 1 \\ \frac{1}{2z^2} & z \geq 1 \\ 0 & \text{otherwise} \end{cases}$$

we define a random variable Y(i) label to indicate if the first model makes significant better prediction for the i-th CRM than the second one (with p-value 0.01):

$$Y(i) = \begin{cases} +1 & p_Z\left(z > \frac{c_1(i)}{c_2(i)}\right) < 0.01 \\ -1 & p_Z\left(z < \frac{c_1(i)}{c_2(i)}\right) < 0.01 \\ 0 & \text{otherwise} \end{cases}$$

To determine if the overall improvement in CC is significant due to one model versus another, we are interested in the p-value of $X = \sum_i Y(i)$. The null distribution of X, D, can be obtained as follows. Under the null hypothesis that the two models have the same predictive ability, for each of the i-th CRM, *Y(i)* is equal to 0 with probability 0.98 and equal to 1 or -1 with probability 0.01 (the value 0.01 comes from the p-value we chose for testing a single CRM). Thus the sum of *Y(i)* follows the distribution produced by a symmetric 1-D random walk where the particle moves in either direction with equal probability 0.01. We are interested in the probability distribution of the particle position after *n* steps (*n* is the number of CRMs), assuming it starts from the origin. This can be calculated from the standard theory of Markov process as $D = P^n v$, where the transition matrix P is defined as

$$P_{ij} = \begin{cases} 0.98 & \text{if } i = j \\ 0.01 & \text{if } |i - j| = 1 \\ 0 & \text{otherwise} \end{cases}$$

and the initial distribution, v, is defined as $v_i = \begin{cases} 1 & \text{if } i = 0 \\ 0 & \text{otherwise} \end{cases}$.

Predicting conserved transcription factor binding sites

We used a recently developed tool, STEMMA (He et al., manuscript in preparation), to predict binding sites in a sequence that are at least partially conserved in orthologous sequences. The idea of this tool is similar to MONKEY [9], except that the binding sites are allowed to undergo lineage-specific changes during evolution, a feature that is becoming commonly recognized [10,11]. STEMMA scores every putative site in a sequence for its likelihood of representing the binding site of some TF, whose PWM is given. The score is determined not only by the match of the site to the PWM, but also by the conservation pattern of the site in orthologous sequences. Specifically, taking a sequence block that matches the length of the PWM, from the multiple alignment, STEMMA assumes that each orthologous site is associated with some functional state (1 if functional and 0 otherwise), and finds the most likely history of functional states in all orthologous sites. In the underlying evolutionary model of STEMMA, the functional state may change over time, modeled by a two-state Markov chain. The sequence evolves according to a neutral model (HKY model, [12]) if the functional state is 0, and according to a constrained

model (Halpern-Bruno model, [13]) if the functional state is 1. Let σ be the history of the functional states along the phylogenetic tree, the best history is found by:

$$\sigma^* = \arg\max_{\sigma} P(\sigma)P(S \mid \sigma)$$

where $P(\sigma)$ is computed from the two-state Markov chain and $P(S \mid \sigma)$ is computed by applying the HB model on the subtree where the functional state is 1 and the HKY model on the neutral subtree. The model parameters are estimated from the sequences using maximum likelihood or taken from the estimates published previously [10,14].

When applying STEMMA in the experiments reported in this paper, we run it in two modes: allowing turnover of sites (as above), or not (i.e. σ is all 1's or all 0's). The multiple alignments of *Drosophila* sequences are taken from the UCSC genome browser.

REFERENCES:

1. Green MR (2005) Eukaryotic transcription activation: right on target. Mol Cell 18: 399-402.
2. Carey M (1998) The enhanceosome and transcriptional synergy. Cell 92: 5-8.
3. Berg OG, von Hippel PH (1988) Selection of DNA binding sites by regulatory proteins. Trends Biochem Sci 13: 207-211.
4. Stormo GD, Fields DS (1998) Specificity, free energy and information content in protein-DNA interactions. Trends Biochem Sci 23: 109-113.
5. Segal E, Raveh-Sadka T, Schroeder M, Unnerstall U, Gaul U (2008) Predicting expression patterns from regulatory sequence in Drosophila segmentation. Nature 451: 535-540.
6. Stormo GD, Fields DS (1998) Specificity, free energy and information content in protein-DNA interactions. Trends Biochem Sci 23: 109-113.
7. Press WH (1992) Numerical recipes in C : the art of scientific computing. Cambridge ; New York: Cambridge University Press. xxvi, 994 p. p.
8. Weisstein EW (2003) CRC concise encyclopedia of mathematics. Boca Raton: Chapman & Hall/CRC. 3242 p. p.
9. Moses AM, Chiang DY, Pollard DA, Iyer VN, Eisen MB (2004) MONKEY: identifying conserved transcription-factor binding sites in multiple alignments using a binding site-specific evolutionary model. Genome Biol 5: R98.
10. Dermitzakis ET, Bergman CM, Clark AG (2003) Tracing the evolutionary history of Drosophila regulatory regions with models that identify transcription factor binding sites. Mol Biol Evol 20: 703-714.
11. Birney E, Stamatoyannopoulos JA, Dutta A, Guigo R, Gingeras TR, et al. (2007) Identification and analysis of functional elements in 1% of the human genome by the ENCODE pilot project. Nature 447: 799-816.
12. Hasegawa M, Kishino H, Yano T (1985) Dating of the human-ape splitting by a molecular clock of mitochondrial DNA. J Mol Evol 22: 160-174.
13. Halpern AL, Bruno WJ (1998) Evolutionary distances for protein-coding sequences: modeling site-specific residue frequencies. Mol Biol Evol 15: 910-917.
14. Moses AM, Pollard DA, Nix DA, Iyer VN, Li XY, et al. (2006) Large-scale turnover of functional transcription factor binding sites in Drosophila. PLoS Comput Biol 2: e130.
